# Determinants of farmers' biosecurity mindset: A social-ecological model using systems thinking

**DOI:** 10.3389/fvets.2022.959934

**Published:** 2022-08-15

**Authors:** Hai-ni Pao, Elizabeth Jackson, Tsang-sung Yang, Jyan-syung Tsai, Watson H. T. Sung, Dirk U. Pfeiffer

**Affiliations:** ^1^Department of Pathobiology and Population Sciences, Veterinary Epidemiology, Economics, and Public Health Group, Royal Veterinary College, Hatfield, United Kingdom; ^2^School of Management and Marketing, Curtin University, Perth, WA, Australia; ^3^Independent Researcher, Taipei, Taiwan; ^4^Department of Finance and Cooperative Management, National Taipei University, Taipei, Taiwan; ^5^Agricultural Bank of Taiwan, Taipei, Taiwan; ^6^Centre for Applied One Health Research and Policy Advice, Jockey Club College of Veterinary Medicine and Life Sciences, City University of Hong Kong, Kowloon, Hong Kong SAR, China

**Keywords:** qualitative study, avian influenza, decision-making, grounded theory, interview

## Abstract

Commercial poultry is often farmed in high-density facilities, therefore, predisposing exposure to threats of infectious diseases. Studies suggest that it is likely that farmers have little motivation to practise on-farm biosecurity. In Taiwan, where high-density intensive poultry production is commonplace, unfortunately, several avian influenza outbreaks have occurred over the past decade despite the establishment of biosecurity procedures. To develop effective interventions, it is essential to understand the determinants of farmers' biosecurity mindset through systems thinking. In this qualitative study, we directly explored the opinions of Taiwan's chicken farmers, and a grounded theory analysis was performed. The study revealed that farmers allocate resources based on their justification for the optimisation of resource utilisation, and biosecurity is the most concerning challenge. Farmers focus on the economic aspects of their production systems, particularly when the implementation of biosecurity increases production costs, and there are multifaceted, complex barriers to implementing on-farm biosecurity. Although the participant farmers accepted to take major responsibility for disease management, paradoxically, some farmers blamed the practicality of government regulations and government employees' attitudes. Additionally, the farmers rejected the government's intentions to ask farmers to take major responsibility for the outbreaks of avian influenza while some of them intended to ignore the perceived risks. Government interventions that were considered not directly related to biosecurity also negatively influenced farmers' willingness to improve biosecurity. Using the interview results together with information in the scientific literature, we constructed a modified six-level social-ecological model to explain the complex influences of macro socio-economic conditions on farmers' biosecurity mindset. The novelty of this research lies in its wider relevance to Taiwan's chicken production industry in that it provides first-hand evidence-based knowledge to demonstrate a wide number of determinants of farmers' biosecurity mindset. This social-ecological model highlights the importance of systems thinking for the development of behavioural interventions and allows adaptation to the local context. The findings of this study have relevance to Taiwan's chicken production industry and potentially to similar systems in other countries in the wider region and should result in more effective animal health management at the farm level.

## Introduction

Global consumption of poultry meats has dramatically increased since the 1970s, and poultry production is expected to increase continuously in developing countries over the coming decade (1). Commercial poultry, especially broilers, can be and often is farmed in high densities (2) even where the ecological environment provided is not favourable.

**Figure 1:**
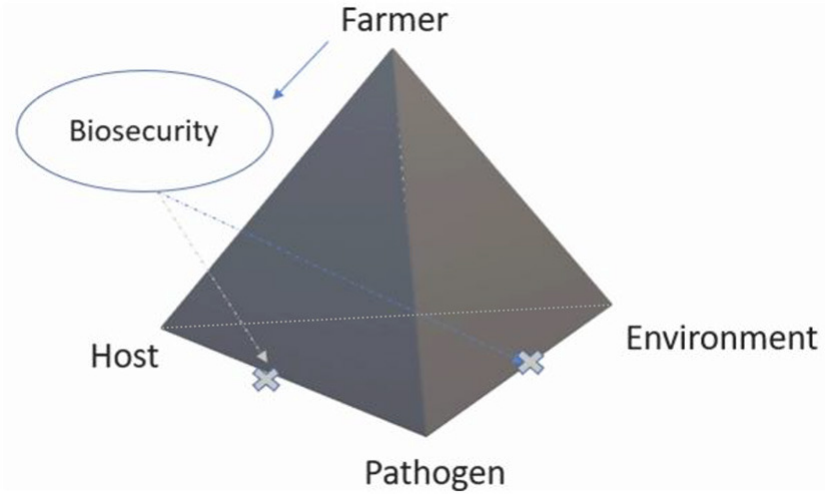
The complexity of factors leading to the success of on-farm biosecurity. The influence of human activity on infectious animal diseases is pervasive; therefore, we constructed a pyramid-shaped representation in which the factors raised by farmers are specifically emphasised. Farmers play a key role in decision-making on the adopting of biosecurity measures to prevent pathogen introduction into a farm or infection of animals.

The epidemiological triad is a conceptual model for the multi-factorial context of disease occurrence that reflects the interactions between host, pathogen and environment. Biosecurity is one of the most effective interventions to prevent and control diseases (Figure 1). Although a “farmer” with appropriate technical expertise and decision-making ability is arguably one of the most important factors influencing the success of biosecurity practises (3), Heffernan et al. (4) suggested that it is likely that even such farmers might still have little motivation to practise effective on-farm biosecurity. Fraser et al. (5) and Laanen et al. (6) also revealed that some farmers posed negative attitudes towards biosecurity. Most biosecurity activities undertaken may not be regulated by laws. If farmers do not comply with biosecurity regulations, government authorities cannot easily observe (or completely monitor) farmers' lack of compliance. Moreover, when governments and the public bear the majority of disease control costs resulting from outbreaks (e.g., compensation), farmers may invest less effort into the prevention of disease outbreaks (7, 8).

Recent studies have identified social and psychological factors that influence farmers' decisions about the implementation of biosecurity such as available information (9), farmers' knowledge and experience (10–13), social pressure (12, 14–16) and time or economic constraints (13, 16–19). This array of factors determines what kind of biosecurity measures farmers will adopt. Table 1 provides a list of a variety of factors that affect farmers' biosecurity mindset as obtained from the literature (References cited are listed in Supplementary Information; Supplementary Table S1). Determinants of farmers' biosecurity behaviours may vary due to different social and contextual environments (20).

**Table 1:** Factors affecting farmers' biosecurity mindset identified in the study together with the literature.

**Level**	**Theme**	**Sub-theme**	
Individual (farmers and chickens)	**Chickens**†		
	**Health and welfare**		
	**Diseases**†		
		Endemic, epidemic or exotic diseases†	
		Disease process	
	**Farmers and farms**†		
	**Attitudes**†		
		Willingness/ Little interest in biosecurity, disease prevention and control†	
		Farmers' attitudes and perceptions towards disease risks (e.g., perceived susceptibility and severity) †	
			*Perception of controllable disease risks*†
			*Luck /fatalism*†
			*Intention to ignore the perceived risks*† (e.g., considering avian influenza a common cold and immunising *chickens by natural infection)*
		Attitudes, awareness, perceptions and beliefs about biosecurity †	
		An underlying failure to appreciate the complex and multiple flows	
		Underlying reasons to appreciate biosecurity†	
			*Perceived benefits*† (e.g., return on investment or maintain business continuity during a disease outbreak)
			*Effectiveness*†
			*Most desirable/useful biosecurity measures* †
			*Feasibility* †
			*Misunderstanding* †
	**Ambition**†		
		Belief in self-efficacy†	
		Ability†	
	**Resources**†		
		Time†	
		Income, capital, or economic concerns†	
		Costs of biosecurity practises†	
		Labour†	
		Land†	
		Access to veterinary service†	
		Testing accuracy†	
	**Trust in the government**†		
		Negative views to information and educational documents provided by the government	
		Negative views to government employees' attitudes†	
		A social dilemma of the trade-off between public benefits and farmers' private interests†	
	**Habits, knowledge and experience**†		
		The available information and sources †	
			*Private veterinarians as the preferred motivators†*
			*The lack of information or education*
		Scientific evidence for the efficacy of biosecurity practises†	
		Current knowledge and experience†	
			*Previous experiences of a disease* †
			*Farmers' knowledge of diseases* †
			*Previous experiences of a specific measure*†
			*Reasonable measures have been done* †
			*Fitting into the current practises*†
	**Social status**†		
		Farming experience†	
		Education†	
		Age†	
		Sex	
	**Characteristics of farmers**		
		Social responsibility for food safety	
		Farmers' personality	
		Guilt, shame and prejudice for disease outbreaks	
		Learning styles	
	**Farm type**†		
		Characteristics of their farms†	
		*Perceived risk to their enterprise*†	
		Farm size†	
		Location†	
	**Workers' aspects**		
		Peer pressure for the accountability and job security/ Manager's commitments to biosecurity/ Education levels/ Personality/ Experiences with disease outbreaks/ Personal beliefs of biosecurity	
Group (family, friends, neighbours and farmers' associations)	**Social culture/ pressure** †		
		Peer pressure	
		Neighbours' attitudes† (e.g., neighbours' attitudes towards farm hygiene and their acceptance of the existing farms)	
	**Industry development**†	
		Cooperation and competition†	
			*Neighbour farmers do not report their outbreaks†*
			*Group membership and group culture†*
			*Negative attitudes about forming groups*
			*Trust in farmers' community†*
			*Peers' knowledge, perceptions and experiences of diseases†*
Organisation (the chicken industry)	**Production conditions**†		
		Costs and profits†	
			*Economic pressure from society and industry* †
		Weather†	
		Agricultural space and environment†	
			*Chaotic and difficult to control*† *(e.g., high density of farms)*
	**Domestic market access**†		
		Consumers' attitudes towards locally-produced chickens†	
			*Consumer-demand* †
			*Consumers' confidence*†
		Access to the domestic market†	
			*Market access channels*†
	**The supply of vaccines and medication**†		
		The access to vaccines†	
		The trust in vaccines and medication†	
	**Industry development**†		
		Organisation culture (e.g. biosecurity culture)	
		The strong power held by relevant stakeholders†	
		Opportunity for the export of chicken meats†	
		Generation gaps and a sunset industry†	
Community (the public)	**Brand establishment to promote local produce**†		
		The lack of trusted domestically produced brands†	
	**Public attitudes towards the poultry industry**†		
		The public's negative attitudes †	
		Unrealistic expectations from the public and the government† (e.g. consumers' expectations for low prices or the government's intentions to ask farmers to take major responsibility for avian influenza outbreaks)	
	**Human health**†		
Government (public policies and government employees' attitudes)	**Government intervention (biosecurity related)** †		
		Compulsory and compensation of biosecurity measures†	
		utility of research †	
			*Biosecurity suggested by scientists lacks common sense and practical experiences*†
			*A simple cost-effectiveness analysis*
			*The lack of trust and credibility in government-related scientific institutions*
			*Not the key issue to study*†
		The lack of regulations or support†	
			*Biosecurity should be compulsory*†
			*Government interventions with financial inducements or penalties*
		The practicality of government regulations†	
			*Opposing attitudes to government intervention* †
			*Negative opinion on control measures* †
			*Biosecurity suggested by the government lacks common sense and practical experiences* †
			*Disadvantages of reporting and dissatisfaction with post-reporting procedures*†
			*Uncertainty and the lack of transparency in reporting procedures* †
		The credibility of biosecurity information provided by the government	
		Negative views to government employees' attitudes†	
	**Responsibility**†		
		Major responsibility belongs to the government or the government should make a greater contribution	
		Major responsibility belongs to farmers†	
	**Government intervention (not directly related to biosecurity)**†		
		The practicality of government regulations†	
		Market mechanisms†	
		The utility of agricultural land†	
Global (international trade)	**Costs and profits**†		
		Feed and petrol†	
	**Industry development**†		
		Opportunity for the export of chicken meats †	
	**Domestic market access**†		
		Competition for the access to the domestic market†	

†Themes/ sub-themes found in this study.

In addition, the application of behavioural change theories in relation to farmers' biosecurity behaviour has increased in recent years (21–23). Behaviour change theories such as the “Theory of Planned Behaviour” (21) and the health belief model (23) have been used to construct the mechanisms of farmers' decision-making with regard to the adoption of on-farm biosecurity based on demographic and socio-psychological factors. However, in public health, the most commonly used theoretical basis of individual-based approaches to study determinants of health behaviour is the social-ecological model (24). McLeroy et al. (25) proposed five levels of factors that influence health behaviour including intrapersonal and interpersonal processes. These multi-level influences may dynamically interact, interrelate or be interdependent across different levels so that the model functions as a whole system. This model explains the interactive relationships between individuals and environments (26, 27). During the COVID-19 pandemic, it has been widely applied to examine multilevel factors influencing COVID-19 preventive behaviours such as mask use, social distancing and vaccine trust (28–31). While Casola et al. (30) argued that an individual's social-ecological network shapes one's decision-making process, Jang (31) further suggested that multilevel efforts can support evidence-based interventions to enhance COVID-19 preventive behaviours.

Human factors are the basis of animal disease control programmes and increase the complexity of animal disease control services and systems (32–34). The influence of human activity on infectious animal diseases is pervasive; therefore, we constructed a pyramid-shaped representation in which the factors raised by farmers are specifically emphasised (Figure 1). Knowledge about these human factors is crucial for increasing the effectiveness of communication and the likelihood of adoption. As French et al. (35) stated, behavioural change interventions based on published studies and/or analyses of demographic and epidemiological data may be not sufficiently effective and feasible. Public sector interventions need to be informed by an adequate understanding of the target groups' motivations, needs and fears as particular biosecurity measures are each farmer's decision. It is necessary to consider the awareness and the incorporation of multilevel interventions for on-farm biosecurity improvement. However, Hidano et al. (36) pointed out that, farmers' behaviours are often considered homogeneous in veterinary epidemiological models. Biesheuvel et al. (20) also demonstrated that most studies have focused on the identification of single-level factors, in particular individual levels, and there is a need to understand how factors at different levels influence farmers' biosecurity behaviours. As such, we used the social-ecological model as the key theory to underpin farmers' mindset in relation to biosecurity behaviours. As a complex web contributes to individual farmer's different biosecurity responses, the social-ecological model can better represent the influence of these complex factors on farmers' biosecurity mindset.

Taiwan is located in a subtropical region that has a hot and humid climate that results in challenging production conditions. As with most other global livestock sectors, economic conditions are becoming more challenging with the increase in the price of animal feed (37). Taiwan joined the World Trade Organisation in 2002 and therefore had to start allowing the import of poultry meats from the United States of America. To reduce the impact of opening the national poultry market to global competition, over the past two decades, Taiwan's government has implemented a series of strategies to support its poultry industry (38) and developed guidelines with biosecurity procedures for disease management and reporting procedures at the farm level. Given that Taiwan's high-density, intensive chicken production system is continuously exposed to infectious disease threats, and poultry products are the second most popular protein source in the country (39), it is essential to examine the views of Taiwan's farmers concerning the challenges they face in relation to on-farm biosecurity so that more effective poultry health management policies can be developed. The lack of existing knowledge on farmers' biosecurity mindset in Taiwan's poultry production system called for this research to understand the unique, under-studied, real-world aspects of the research environment. As such, the key objective of this study was to construct determinants of Taiwan's farmers' biosecurity mindset, with a particular focus on the impact of systemic problems on the complexity of factors with the constructs of the social-ecological model.

## Materials and methods

**Figure 2:**
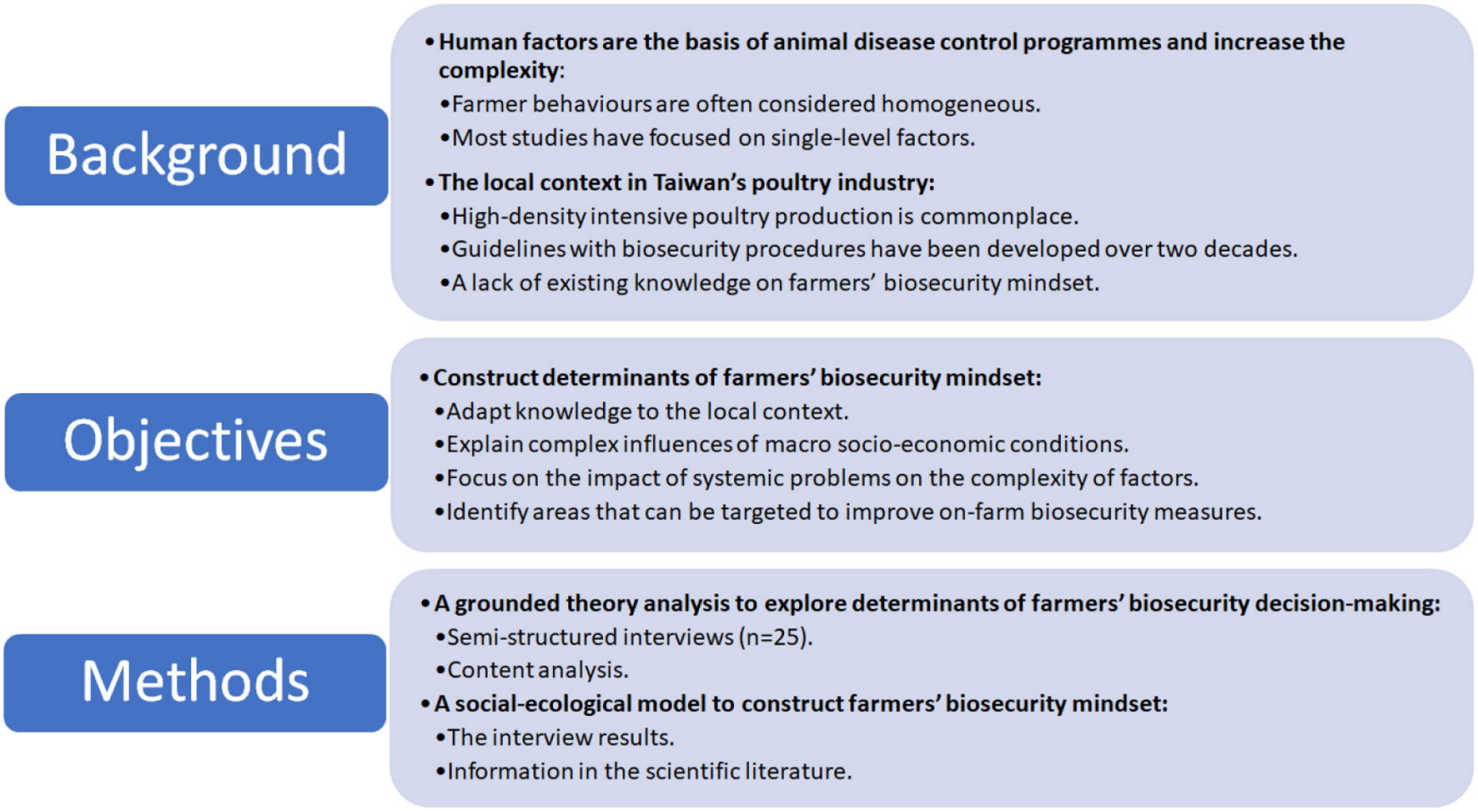
The framework of the study. The opinions of Taiwan's chicken farmers were directly explored through semi-structured interviews, and a grounded theory analysis was performed. By integrating themes and sub-themes revealed in this study together with the literature, a modified six-level social-ecological model was constructed to explain the complex influences of macro socio-economic conditions on farmers' biosecurity behaviours.

Considering the scarcity of the literature on this subject with regard to Taiwan and the wider region, exploratory research was needed to enable the attitudes and perceptions of participants to further develop the limited nature of existing knowledge. Likewise, a grounded theory approach was chosen to acknowledge the nuances of participants' knowledge and gain authentic insight into the research context. The limited existing knowledge of biosecurity activities amongst Taiwan farmers meant that findings grounded in the data were unique to the study and not unduly influenced by speculation. Figure 2 presents the framework of the study.

### Interviews and data analysis

An interview guide (Supplementary Information SI.; Appendix 1: Interview guide) was developed and used to probe the participants to express their experiences and views through telling their stories in relation to their farm management and on-farm biosecurity. Following approval by Ethics and Welfare Committee of the Royal Veterinary College, University of London, the United Kingdom (approval #URN 2014 0116H) (approval #URN 2014 0116H), the two-page interview guide was administered to farmers to gather farm management information, including on production and biosecurity practises. The topics related to farm type, biosecurity practises, resources, the surrounding environment and farmers' experience in farm management. Interviews comprised of simple and short open-ended questions to minimise potential risks of misunderstanding and maximise respondents' opportunity for freedom of expression (40, 41).

Participating farmers represented the three main chicken production sectors in Taiwan, including egg farms (EF), white-chicken broiler farms (WB) and indigenous chicken farms (IC). Egg farmers are commercial-scale egg producers, predominantly using the Hy-Line layer breed (42). White-chicken broiler farms are meat-producers using Avian, Arbour Acre, Hubbard and Rose broiler breed strains (43) while indigenous chicken farms are meat-producers of chickens using domestic breeds (39). Each of these production sectors caters to a different market in Taiwan.

The interviewees were recruited through a variety of routes. The local livestock disease control centre (LDCC), the Poultry Association and private feed companies identified suitable chicken farmers for on-farm interviews. Recommended potential participants were recruited by telephone and provided with a detailed explanation of the study. There is likely to be selection bias in that the sample overrepresented individuals with a higher level of chicken farming standards. The interviewer visited chicken farms at a time convenient to farmers and was accompanied by officials from the LDCC or members of the Poultry Association in an effort to gain the farmers' trust. The interviews took the form of conversations so the participants could raise what they considered to be relevant to the topics. Guided by the work of Flick (44), Jovchelovitch and Bauer (45), and Berg (46), the interview started with questions that elicited responses such as ‘Can you tell me some things about yourself? How long have you or your family been involved in this farm? What makes it difficult to be a farmer? Have you faced any big challenge/ disaster that happened during the past 2 years?’. These questions encouraged farmers to tell their experiences in farming and, most importantly, the challenges of farming. The farmers first addressed the broad question about the barriers when raising chickens. Then, a more focused question such as ‘the barriers when implementing on-farm biosecurity’ was discussed. The interview took approximately 1.5–2 h to complete. Participants were assured that all the data would be anonymous and stored securely.

Most people in Taiwan have traditional Chinese social and cultural values, emphasising the importance of order, harmony, tolerance and forgiveness. Based on these values (47, 48), verbal (rather than written) consent was obtained to reduce each participant's concerns in relation to anonymity. Semi-structured interviews were conducted in Mandarin and translated into English. Any information which could potentially result in compromising an interviewee's anonymity was removed from the transcripts. The data were analysed by qualitative content analysis (40, 49, 50). Open coding of respondents' responses was performed to determine major and minor themes. As recommended by Elo and Kyngäs (40) and Creswell (51), the processes were reviewed to refine the themes and improve the validity and reliability of the data. Moreover, codes were cross-checked by comparing results from the literature.

### Theoretical construct for the social-ecological model

The social-ecological model served as the key theory to underpin farmers' mindset in relation to biosecurity behaviours. This study was framed by adopting the social-ecological model of McLeroy et al. (25). Although empirical research to guide model development is limited, the model constructed was tailored to suit the biosecurity behaviours of the study group. On the basis of factors identified in this study, the extensive scope of key elements in relation to farmers' biosecurity adoption was required to build up meaning. This model incorporated multiple determinants into different levels of influence on biosecurity behaviour by taking into consideration the interaction of biosecurity behaviours across these different levels of influence.

### Integration of empirical evidence with the literature

The purpose was to systematically understand drivers associated with individual farmers' on-farm biosecurity attitudes and behaviours with a particular focus on the impact of systemic problems in relation to the complex interrelationships between factors. As biosecurity is of wide relevance for many types of livestock production, current research about the livestock industry relating to farmers' biosecurity attitudes, behaviours and relevant drivers were integrated into the social-ecological model developed in this research for confirmation of the underlying factors.

## Results

Twenty-five farmers were interviewed, at which point theoretical saturation was reached (52, 53). The main form of chicken farming in Taiwan is family production with males being the primary decision-makers (54). Despite the researchers' best efforts to source a more diverse sample, only males were willing to participate in this study as family-owned businesses are the most common business type in Taiwan. Before conducting each interview, the interviewee was requested to reaffirm that he was the farm's leading decision-maker. The sample is characterised as consisting of only males (*n* = 25) with more than 20 years of farming experience (Supplementary Material SI.; Supplementary Table S2). Amongst the participants, 13 farmers were local group leaders of the Poultry Association.

Themes and subthemes emerging from the interview data are summarised in Table 2. Data were contextualised by using codes to identify individual farmers and related farm types (55). The study explored multi-faceted barriers from farmers' viewpoints to understand potential challenges of farm management in relation to on-farm biosecurity. Farmers' interviews suggest that biosecurity is a part of farm management; that is, when considering the adoption of biosecurity measures, they must adjust resource allocation and utilisation based on resource constraints and the balance of resource demand and supply availability. Seven main barriers to farming were revealed and explored in more detail: production conditions (21/25); government interventions (14/25); domestic market access (12/25); industry development (11/25); farmers' ambition to expand their business (7/25); social culture (4/25); and on-farm biosecurity (22/25). Table 3 shows example quotes of themes and subthemes.

**Table 2:** Summary of themes emerging from the interview data [by percentage (%) of participants who addressed them].

**Themes/Subthemes**	**%**	**Themes/Subthemes**	**%**	**Themes/Subthemes**	**%**
**I. Production conditions**	**84**	**III. Domestic market access**	**48**	**VII. On-farm biosecurity**	**88**
1.Costs and profits†, §, ‡‡	72	1. Farmers' access to the domestic market supply chain§,‡‡	32	1.Infectious diseases of chickens†	60
				a. Avian influenza	60
2.Weather§	28	2. Brand establishment to promote local produce¶	20	b. Salmonellosis	12
				c. Infectious bronchitis	8
3.High density of farms§	20	3. Consumers' attitudes towards locally-produced chicken§	20	d. Coccidiosis	8
				e. Infectious bursal disease	4
				f. Newcastle disease	8
				g. Chronic respiratory disease	4
**II. Government interventions (excluding on-farm biosecurity)**	**56**	**IV. Industry development**	**44**		
1.Market mechanisms † †	32	1. Collaboration and competition between farmers‡	32	2.The government regulations and policy related to on-farm biosecurity† †	56
2.Government employees' attitudes to the chicken industry and farmers † †	12	2. The strong power held by relevant stakeholders§	24	a. The practicalities of government regulations & policies	48
				*Paper trays for egg packaging*	28
3.The utility of agricultural land † †	8	3. Opportunity for the export of chicken meats§, ‡‡	12		
				*Anti-bird netting*	24
				*Contracted veterinarians*	16
		4. Generation gaps and a sunset industry§	6	*Compensation for stamping out measures*	8
4.The practicalities of government regulations and policies † †	32	**V. Farmers' ambition to expand their business**†	**28**		
a. Slaughter ban	32	**VI. Social culture**	**16**		
b. Drug residue	8	1. Public attitudes towards the chicken industry¶	12	b. The utility of research	12
		2. Neighbours' attitudes towards the farms‡	8	c. The lack of regulations	12
				3.The supply of vaccines and medication§	16
				a. The access to vaccines	12
				b. Trust in vaccines and medication	8

†The individual-level; ‡The group-level; §The organisation-level; ¶The community-level; † † The government-level; ‡‡ The global-level.

**Table 3:** Example quotes of themes and subthemes.

**Theme**	**Subtheme**	**Example quotes**	**Farm code**
**Production conditions**			
	1.Costs and profits	*They [farmers] want to use the minimum amount of feed to convert the maximum amount of meats by shortening the feeding period. […] Normally, biosecurity measures, particularly vaccines, count for 5-8% of the total cost. In my case, it is 5%*.	WB, a local leader of the Poultry Association, Interview 1
	2.Weather	*The occurrence of diseases is often associated with seasonal changes. Temperature changes cause infections, often making a lot of chickens get sick, and even die*.	WB, Interview 3
	3.High density of farms	*You should go to Changhua, and you will know why avian influenza cannot be controlled. Such a high-density farming area. […] To be honest, there is a lot of progress in the prevention and control of avian influenza on good farms [in Taiwan]. But in Changhua, avian influenza is really a big problem and we can hardly control it*.	EF, a local leader of the Poultry Association, Interview 2
	4. Generation gaps and a sunset industry	*I've raised chickens for 40 years, but my son doesn't want to take on my business. My investment in improving facilities is a waste even though I recognised that the use of modern evaporative cooling systems can improve biosecurity*.	IC, Interview 7
**Government interventions (excluding on-farm biosecurity)**			
	1.Market mechanisms	*Ignorant consumers also think the government should have to balance prices. […] Without a reasonable increase in egg prices, it is unlikely that we will be able to cover more costs to do more biosecurity*.	EF, a local leader of the Poultry Association, Interview2
	2.Government employees' attitudes to the chicken industry and farmers	*They behave like the proverb: “Do more, wrong more; do less, wrong less; do nothing, nothing wrong”*.	IC, a local leader of the Poultry Association, Interview 6
	3.The utility of agricultural land	*Taiwan's agricultural land is insufficient; however, the government still allows the speculation of agricultural land to exist in Taiwan*.	EF, a local leader of the Poultry Association, Interview 2
	4.The practicalities of government regulations & policies		
	a. Slaughter ban	*The policy has great impacts on our livelihoods, including the reduction of chicken numbers*.	IC, a local leader of the Poultry Association, Interview 2
	b. Drug residue	*We got a false-positive result for drug residue tests, but this was caused by cross-contamination via feed transportation cars. I think the system is unreasonable because our business was affected by this false-positive case […] There is no effective way to help us rebuild our reputation*.	EF, a local leader of the Poultry Association, Interview 3
**Domestic market access**			
	1.Farmers' access to the domestic market supply chain	*Egg prices will be maintained at between $25 to $30 New Taiwan Dollars (NTD), which is the market mechanism. People who have domestic market access in the hand will win. […] It is only when we have revenue that we can consider how to improve our farms*.	EF, a local leader of the Poultry Association, Interview 3
	2.Brand establishment to promote local produce	*I want to establish my own brand. But because of marketing costs, it is impossible for me to do that. […] I have to stick with the current mode of operation. I do not want to invest too much money*.	EF, Interview 7
	3.Consumers' attitudes towards locally-produced chicken	*If our consumers could know that paying more can ensure food safety, they will be willing to spend the money. […] Without consumer support for higher egg prices, farmers will not be willing to invest in improving food safety or on-farm biosecurity*.	EF, a local leader of the Poultry Association, Interview 2
**Industry development**			
	1.Collaboration and competition between farmers	*Because of the competitive pressures, I have to seek more creative management strategy. [...] I work 365 days a year without a break. I have done all my best*.	EF, a local leader of the Poultry Association, Interview 3
	2.The strong power held by relevant stakeholders	*Breeder farmers integrate together to control the total number of chicks, avoiding excessive quantity in the market, but the price for a chick has increased to $20 NTD from $4 NTD. It is now $25 NTD. [...] This is unfair competition. [...] It is a monopoly. [...]How can we have the funds to improve on-farm biosecurity when we have less income?*	WB, a local leader of the Poultry Association, Interview 1
	3.Opportunity for the export of chicken meats	*Export is a kind of opportunity, but it may only happen to breeder chickens for the export. The export of breeder chickens is not necessarily helpful to the whole industry unless we can export chicken meats. [...] As long as we can export chicken, farmers will be motivated to meet international standards*.	IC, a local leader of the Poultry Association, Interview 6
**Farmers' ambition to expand their business**			
		*The objective of my enterprise is not to be the first but to be unique. [...] I have already established my brand. My farms have already adopted an automated way to raise red-feathered chickens and I already have sales partners. I expect my revenue to increase year on year*.	IC, Interview 3
**Social culture**			
	1.Public attitudes towards the chicken industry	*The political environment is not friendly to agriculture, including the chicken industry. [...] If the society does not recognise our efforts, why should we improve the chicken farming environment?*	EF, a local leader of the Poultry Association, Interview 5
	2.Neighbours' attitudes towards the farms	*I would like to change my rearing style into grazing in the future, but, because there are communities around my farms, they will complain about it. I'm afraid of neighbours' complaints about the bad smell*.	IC, Interview 1
**On-farm biosecurity**			
	1.Chicken diseases	*1. Bird flu will happen definitely, but we should not allow zoonotic types to occur*.	EF, a local leader of the Poultry Association, Interview 2
		*2. Chickens are our property and I will lose money if any chicken dies*.	WB, Interview 6
	2.The government regulations and policy related to on-farm biosecurity		
	a. The practicalities of government regulations & policies		
	*Papertrays* for egg packaging	*1. Egg farmers are not willing to pay money out of their pockets if the government does not agree to increase egg prices once they use paper trays*.	EF, a local leader of the Poultry Association, Interview 9
		2. *Who should pay it? It began with contradiction. As a consumer, you said I bought your eggs, why should I pay? But I was the producer. I sold goods, but why should I give you money for this? […] I shall make money, and this is my profit. Profit is fundamental and nobody wants his money to be taken away. May I ask you who is right? “*	EF, a local leader of the Poultry Association, Interview 2
	Anti-bird netting	*The government's anti-bird netting is difficult to instal, although the government asks us to do that. […] Poor outcomes. When we actually operated it, there still were some birds coming in, but they had no way to go out*.	EF, Interview 1
	Contracted veterinarians	*Having a contracted veterinarian is only for the purpose of obeying the policy. We do not have money to employ veterinarians. [...] We seek for the veterinarian's assistance only when we need a final diagnosis*.	WB, a local leader of the Poultry Association, Interview 1
	Compensation for stamping out measures	*AI outbreaks accelerated the deterioration of the farmers' opposition to the government. […] In fact, in 2012, there was an area with HPAI outbreaks in Changhua County, and the government stamped out the entire area. But the government did not consider how farmers could survive without selling chickens. After culling, the farmers had to stop rearing for 6 months, and then waited for another 2 months for the use of sentinel chickens. It required 8 months without any selling. So how do you expect the farmers to keep their living?*	WB, a local leader of the Poultry Association, Interview 1
	b. The utility of research	*The studies are not the one our professors should study, not the key issue we concern*.	WB, a local leader of the Poultry Association, Interview 8
	c. The lack of regulations	*If the government does have interests in biosecurity, they should promulgate the provisions related to on-farm biosecurity measures*.	EF, a local leader of the Poultry Association, Interview 2
	3.The supply of vaccines and medication	
	a. The access to vaccines	*HPAI is a threat to humans and a lot of chickens will be dead, but there is no vaccine available for HPAI*.	EF, a local leader of the Poultry Association, Interview 3
	b. Trust in vaccines and medication	*In 2008–2009, there was a pandemic of infectious bronchitis because of attenuated vaccines or insufficient subculture of vaccines. […] 7–8 years ago, there were serious outbreaks of coccidiosis because cheaper Chinese-made drugs had problems*.	WB, a local leader of the Poultry Association, Interview 1

**Figure 3:**
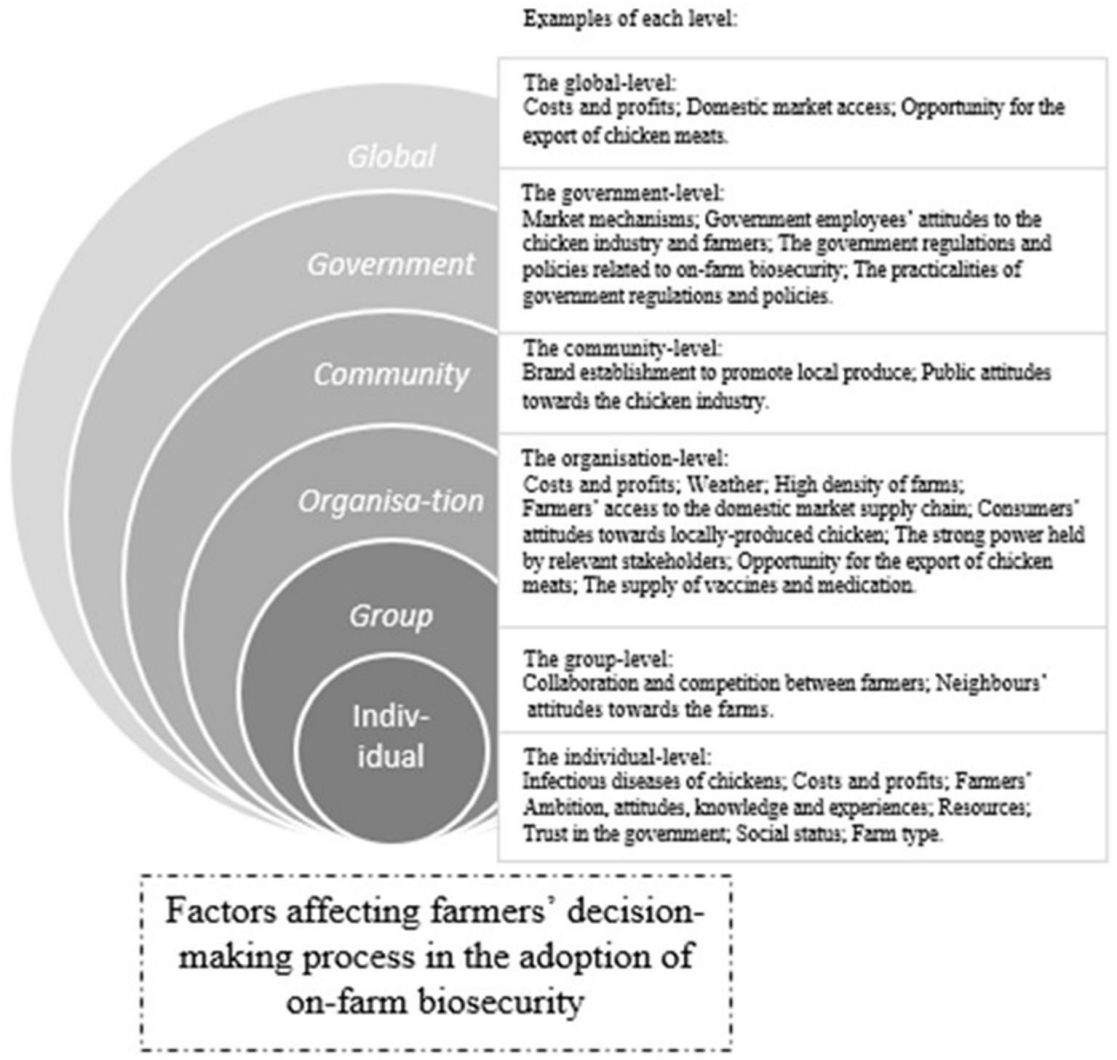
An exploratory social-ecological model of factors affecting farmers' decision on biosecurity. A modified six-level social structure was constructed to explore farmers' decisions for the implementation of biosecurity at farm level. The model explains the complex influences of macro socio-economic conditions on farmers' biosecurity behaviours; global effects were also included.

On the basis of the seven key elements identified in the study, a modified social-ecological model was constructed to demonstrate the complex interactions between factors at different levels from individual farms to the global community. Although a five-level model was proposed by McLeroy et al. (25), a modified six-level social structure was constructed in this study to explore the interactive structure between the different societal layers from individual farms to the global community with regard to on-farm biosecurity behaviours (Figure 3 and Table 1). The levels of influence in the social-ecological model lead to the conceptualisation of potential interventions aimed at behaviour change. In addition, Table 1 and Supplementary Table S1 show the themes and sub-themes revealed in the current study together with those described in the literature such as (1) “Intention to ignore the perceived risks” under the theme of “Attitudes” and “Government employees” attitudes” under the theme of “Trust in the government” at the individual level; (2) “Neighbours” attitude” under the theme of “Social culture/ pressure” at the group level; (3) “Market access channels” under the theme of “Domestic market access” together with “The strong power held by relevant stakeholders”, “Opportunity for the export of chicken meats” and “Generation gaps and a sunset industry” under the theme of “Industry development” at the organisation level; (4) “Major responsibility belongs to farmers” under the theme of “Responsibility” together with “The practicality of government regulations”, “Market mechanisms” and “The utility of agriculture land” under the theme of “Government intervention” at the government level; (5) “Feed and petrol” under the theme of “Costs and profits”, “Opportunity for the export of chicken meats” under the theme of “Industry development” and “Competition for the access to the domestic market” under the theme of “Domestic market access” at the global level.

### Individual-level (farmers and chickens)

The individual-level included factors affecting the farmers' adoption of biosecurity measures on the farms. There are two groups of factors: (1) Chickens: “Infectious diseases of chickens” is one of the most important factors affecting the adoption of biosecurity measures indicated by farmers in this study. Valeeva et al. (56) and Moya et al. (16) suggested the spread potential of infectious diseases (e.g., endemic, epidemic or exotic diseases) will affect farmers, risk perceptions and behaviours towards animal disease management. Moreover, Heffernan et al. (4) indicated that “the disease process” will also affect farmers” biosecurity behaviours. However, although farmers in the study agreed that biosecurity keeps chickens healthy, none of them mentioned animal welfare when considering the implementation of biosecurity; (2) Farmers and farms: as shown in Table 1, there are nine themes and a wide number of associated subthemes related to farmers” decisions about the implementation of biosecurity measures such as “Intention to ignore the perceived risks” under the theme of “Attitudes” and “Government employees” attitudes” under the theme of “Trust in the government”.

Almost all farmers (24/25) develop their biosecurity strategies to decrease the risks of disease outbreaks. Most farmers expressed concerns about the chicken disease (15/25), and the most concerning disease on farms was avian influenza (15/25). While 15 farmers were concerned about avian influenza outbreaks as ‘*HPAI is a threat to humans and a lot of chickens will be dead*’ *(WB, a local leader of the Poultry Association, Interview 1)*, 10 farmers regarded avian influenza in chickens as similar to a common cold in humans. Seven farmers indicated their concerns about disease outbreaks, with temperature variation exacerbating the situation. In contrast, there was one farmer who negated the effectiveness of biosecurity measures because he considers preventing disease transmission from neighbouring farms to be impossible. He indicated that the most risk is born from situations beyond their control: neighbours do not report their outbreaks including suspected cases. In addition, 12 farmers frequently stated that high input costs such as imported feed materials and fuel as well as a lack of labour force and time are major concerns that influenced their motivation to improve on-farm biosecurity. With clear evidence from farmers that “*Chickens are our property and I will lose money if any chicken dies.” (WB, a local leader of the Poultry Association, Interview 1)*, most farmers are willing to implement a certain amount of biosecurity measures.

“*Young or educated farmers will attend seminars or discuss with others to improve their knowledge about biosecurity. Local groups and the local Poultry Association will arrange training courses. But it depends on farmers' willingness. If they care, they definitely will attend relevant classes. [...] It is polarised.” (WB, a local leader of the Poultry Association, Interview 1)*

Almost all farmers (24/25) used vaccines to prevent a number of infectious diseases, including Newcastle disease, infectious bursal disease and so on. All of them felt satisfied with their vaccination strategy as “*I am not afraid of any disease because of intensive vaccination*” *(WB, a local leader of the Poultry Association, Interview 5)*. In addition, they all believed that their hands-on farming experience provided them with the necessary expertise to determine a chicken's health condition and to decide on relevant vaccination and medication programmes as “*experiences are very important” (IC, a local leader of the Poultry Association, Interview 4)*. A farmer indicated that, as most farmers have personal experience with avian influenza outbreaks on their farm (or shared by other farmers) together with required knowledge of avian influenza and biosecurity, he believes that they have implemented the most relevant biosecurity measures that are compatible with their current husbandry practises.

Most farmers believed their experiences are trustable: “*we know what we should do and I do not need government employees without experience to tell me what or how we should do” (EF, Interview 10)*. The sentiment “*government officials are not willing to understand our needs.” (IC, a local leader of the Poultry Association, Interview 6)* also reflects that those farmers distrust government employees' sincerity and interest. The view that “*mandatory biosecurity measures should avoid unnecessary impacts on their routine farm management*” *(WB, a local leader of the Poultry Association, Interview 2)* was emphasised by eight farmers. This implies that there are hindrances to changing farmers' current practises. Twelve farmers expressed a negative attitude towards the effectiveness of mandatory biosecurity control measures, especially in relation to anti-bird netting, and five farmers felt that avian influenza was uncontrollable (fatalism) due to the high-density of farms and the variation of virus strains as “*there is no way to prevent any disease, especially avian influenza*” *(EF, Interview 1)*. One farmer tended to let the chickens get natural infection:

‘*By reducing the frequency of disinfection and cleaning as well as increasing their contact with the environment. Because I use natural grazing, the disease is coexistent with chickens. [...] Bird flu is just a cold, why so serious?’ (EF, a local leader of the Poultry Association, Interview 3)*

Five farmers, especially egg farmers and indigenous chicken farmers expressed their desire to establish their own brand. Seven farmers developed their management and biosecurity strategies, expanded their farm size and “*began to gain a sense of accomplishment*” *(EF, a local leader of the Poultry Association, Interview 2)*. As such, the theme of “farmers' ambitions to expand their business” emerged from the data.

### Group-level (family, friends, neighbours and farmers' associations)

The group-level explored the close relationships within social circles, for example, farmers' family members and friends as well as farmers' associations and neighbours, which are likely to affect individual farmers' biosecurity behaviours through the sharing of attitudes, experiences and resources. Barclay (57) indicated that farmers, who perceived disease risks as not controllable, are less likely to implement biosecurity measures. Studies revealed that little trust in their community or other farmers will negatively influence farmers” adoption of biosecurity measures (4, 58). Heffernan et al. (4) further suggested that although some farmers expressed negative attitudes towards forming groups, some farmers sought information from other farmers. There are two themes and three subthemes related to farmers' decisions about the implementation of biosecurity measures, as shown in Table 1, such as “Neighbours' attitude” under the theme of “Social culture/ pressure” and “Cooperation and competition” under the theme of “Industry development”.

Establishing good relationships with neighbours is a common desire of most farmers, as it may affect their ability to manage their farms in the future. However, two farmers still reported problems as they are afraid of neighbours' complaints and often encounter unfriendly responses such as their neighbours' refusing to accept the existence of the farms. Farmers also felt their neighbours' complaints about noise and odours are unfair, especially when they keep chickens in chicken houses and use disinfectants twice a day. Two farmers worried that the government cannot ensure that neighbours do report their outbreaks, particularly suspect cases. They emphasised that, due to the high density of farms without appropriate government control, farmers might lack the motivation to implement better biosecurity measures.

All farmers were members of the local poultry associations as well as other local poultry farmer groups. Only one farmer did not attend relevant education programmes because of lack of time. This farmer expressed his attitude towards avian influenza and that he doubted the efficacy of disinfection (with misunderstandings).

‘*I feel it is very hard to prevent bird flu. I heard and saw from other farmers' experiences. […] We have insecticidal measures, but there is no routine disinfection measure. I feel that disinfection has little effect.’ (EF, Interview 1)*

Most farmers felt satisfied and appreciated what they have learned about biosecurity. Twenty farmers felt confident about what they have done with regard to on-farm biosecurity:

‘*I've done a lot; thus, I do not worry about avian influenza disease (AID)’*. *(WB, Interview 6)*

‘*The more courses they provide, the better I can learn. The private feed companies and the Poultry Associations will offer free training courses in relation to biosecurity. [...] Veterinary schools will also notify us to attend their courses.’ (IC, Interview 1)*

However, three farmers complained that current research does not meet their needs, as ‘*biosecurity is described as wonderful by scholars, but it is not like what we experience in reality.' (EF, a local leader of the Poultry Association, Interview 2)*.

### Organisation-level (the chicken industry)

The organisation-level examined the chicken industry, in which social relationships, resource supply and production condition issues occurred that are associated with adopting biosecurity measures. While Ellis-Iversen et al. (59) and Enticott and Wilkinson (60) indicated that farmers' biosecurity decisions are influenced by economic pressure and organisation culture from society and industry, Barclay (57) and Lestari et al. (61) suggested that weather and agricultural space/environment can affect the implementation of biosecurity. Four themes identified in the interviews, as shown in Table 1, including “Production conditions”, “Domestic market access”, “The supply of vaccines and medication” and “Industry development”, are associated with the adoption of biosecurity measures at the organisation-level.

Farmers explained that the future of their industry is influenced by numerous, complex and multifaceted factors. Six farmers often referred to the concerns with respect to “generation gaps” and “a sunset industry”. Because “*young people do not want to raise chickens anymore” (IC, Interview 1)'*, older farmers did not want to invest in their farms. For example, although building evaporative cooling systems would allow coping better with temperature and ventilation problems as well as improve biosecurity, those farmers preferred not to upgrade their farm facilities. In addition, farmers with open chicken houses mentioned that “*productivity is poor when encountering hot and humid climates in summer or cold climates in winter*” *(EF, Interview 8)* and indicated their concerns with respect to disease outbreaks being caused by temperature change, making the situation worse. Five farmers expressed their concerns about the high density of farms. It was stated that this threat encourages farmers to enhance their on-farm biosecurity.

Farmers frequently stated that the high costs of imported feed materials and petrol are serious concerns. Such concerns about resource supply negatively affect their willingness to invest in biosecurity. To avoid losing money, farmers sometimes “*reduce breeding numbers, in case chickens cannot be sold out*” *(WB, Interview 3)*. During the growth period of chickens, farmers use various strategies to maximise profits by taking into account specific characteristics, such as shortening the feeding period.

“*Farmers pursue the rapid growth of chickens. They want to use the minimum amount of feed to convert the maximum number of meats by shortening the feeding period. [...] Some farmers prefer to sell chickens on the 35th day to maximise their business profits. [...] Farmers having more batches per year will win the competition.” (WB, a local leader of the Poultry Association, Interview 1)*

In response to strong competition in the domestic market from imported meats, five white-chicken broiler farmers appeared to have more confidence in adapting to the challenges, while three indigenous chicken farmers worried that “*ageing farms will be eliminated*” *(IC, a local leader of the Poultry Association, Interview 5)*. With contributions from well-established strategic alliance systems, white-chicken broiler farmers have established better collaboration with other farmers that allows them to respond to any change in supply and demand in the domestic market; meanwhile, their farmer associations also have published requirements for the implementation of on-farm biosecurity in case of the implication of any exotic disease invasion with the imports of chicken meats.

Six white-chicken boiler and egg farmers discussed the perceived “monopoly” of relevant stakeholders–the strong power held by relevant stakeholders: breeder farmers control the number of day-old chicks, resulting in the control of chick prices. In addition, feed companies play vital roles in the cost management of the value chains, especially to contracted white-chicken broiler farms. At the same time, slaughter plants determine the marketing channels of indigenous chicken farms. Those control mechanisms affect the development of the industry as a whole, which in turn affects the income of farmers and their willingness to invest in biosecurity.

Five farmers indicated that they struggle to maintain local market access for their products, and three farmers admitted that they worry about their competitiveness in the future. Thinking in “commercial” terms, rather than “production” terms, emerged as a critical theme, particularly among egg farmers. Further, with regard to access to the domestic market supply chain, they indicated the challenges they encounter such as the price competition were due to consumers' expectations for low prices. Egg farmers seem to be more willing to accept (possibly due to better insight) the importance of consumers' attitudes towards domestic market access.

After avian influenza outbreaks since 2004, there have not been any exports of chicken and chicken products. Taiwan's chicken industry dramatically declined due to this loss. Regaining export market access will lead to industrial upgrading. However, three farmers believed that it is difficult for Taiwan's chicken industry due to insufficient disease control such as avian influenza or quality control such as drug residue. In addition, farmers also indicated their worries about there being no vaccination for the prevention of avian influenza and ineffective vaccines for other relevant infectious diseases based on their past experiences with infectious bursal disease outbreaks.

### Community-level (the public)

The community-level revealed the broad societal factors related to the public that created the culture in which on-farm biosecurity is encouraged (or inhibited). As also reported by Ellis-Iversen et al. (59), intrinsic barriers such as non-supportive social norms and extrinsic barriers from culture and society will affect farmers' willingness to implement disease control programs. Interestingly, although farmers in the study recognised that avian influenza is a zoonosis, none of them mentioned their own health when considering the implementation of biosecurity. Three themes and three associated subthemes related to farmers' decisions about the implementation of biosecurity measures were discovered as shown in Table 1, such as “The lack of trusted domestically produced brands” under the theme of “Brand establishment to promote local produce” and “The public”s negative attitudes' under the theme of “Public attitudes towards the poultry industry”.

Due to various restrictions, including marketing costs, relationships with stakeholders and production capacity, five farmers felt difficult to realise their ambitions to establish their own brands. They also felt that the public's expectations for low prices were unrealistic. Two farmers also worried that consumers' confidence in locally-produced chicken was affected by a series of food safety scandals in Taiwan.

In addition, three farmers indicated that the value of agriculture is not recognised by the public as “*the political environment is not friendly to agriculture, including the chicken industry*” *(EF, a local leader of the Poultry Association, Interview 5)* and “*Taiwan's people only recognise and appreciate high technology enterprises because they improve Taiwan's economy*” *(WB, Interview 7)*.Due to avian influenza outbreaks, there was distrust towards farmers and their poultry products amongst some consumers. Those farmers who looked at avian influenza in chickens as a common cold rejected the government's intentions to ask farmers to take major responsibility for avian influenza outbreaks:

“*People have chances to catch colds, but we want to reduce the chance. As I took care of my child very well, he still caught a cold. Tell me what I can do? We are talking about biosecurity, if you can keep your child away from sickness for a decade, then we can criticise whether the others are good or bad. If you cannot take good care of your son, how can you blame our farmers who have to take care of million chickens? It is unfair to say that our biosecurity is not good. All animals will get sick. […] It is just a panic of bird flu. People will walk around, but the chickens stay there. We have tried to control it, but how can you expect us to do much better?” (EF, a local leader of the Poultry Association, Interview 2)*

### Government-level (public policies and government employees' attitudes)

The government-level represented the importance of public policies and the government employees' attitudes that assist in the implementation of on-farm biosecurity. These factors cover a wide range of current government interventions, including biosecurity-related or non-related issues, and are strongly associated with farmers' willingness to implement on-farm biosecurity. While farmers in Australia and the UK suggested that the major responsibility of biosecurity belongs to the government or government should make a greater contribution (57, 62), Taiwan's farmers accept that “*farmers shall take the major responsibility to implement biosecurity*” *(WB, Interview 6)*. They thought the effectiveness of biosecurity is determined by farmers' attitudes and precaution measures. Three themes and 10 subthemes related to farmers' decisions about the implementation of biosecurity measures were identified, as shown in Table 1, such as “Not the key issue to study” under the theme of “Government intervention (biosecurity related)”; “Major responsibility belongs to farmers” under the theme of “Responsibility”, “The practicality of government regulations”, “Market mechanisms” and “The utility of agriculture land” under the theme of “Government intervention (not directly related to biosecurity)”.

Three farmers widely associated the function and performance of government interventions with government employees' attitudes. In addition, 15 farmers indicated specific examples to show the impracticalities of government regulations and policies:

(1) The government imposed a slaughter ban in 2013 to combat the problem of H7N9 avian influenza. Eight indigenous chicken farmers took the slaughter ban as being an example that ‘the policy has a great impact on our livelihoods, including reduction of chicken numbers’ (IC, a local leader of the Poultry Association, Interview 2) to express their frustration.
(2) Seven egg farmers indicated that they support the proposal by the government of using paper trays to improve the sanitation of egg packaging; however, they are not willing to do it if the government does not agree to increase egg prices. Farmers also mentioned other problems arising due to not being ready for a paper tray policy, as ‘*Paper egg trays cannot be stacked for transportation. They pose threats to the environment. They are not suitable for the current mode of transportation.’ (IC, a local leader of the Poultry Association, Interview 6)*.
(3) In relation to the control of HPAI, all participants were aware of the need for farmers and veterinarians to report suspect cases immediately. However, four farmers indicated that difficulties are preventing them from following the policy, including the amount of compensation and the time it takes until they receive it.

Market prices affect profitability and farmers' willingness to invest in biosecurity. Five farmers frequently referred to the government's lack of understanding in relation to market mechanisms; however, three farmers preferred for Taiwan's government to implement stronger interventions to control chicken prices. Farmers sometimes express a need to upgrade technology, reduce costs and ease competitiveness. Five farmers indicated research and development of vaccines and medication needs to be prioritised.

When expressing their concerns and difficulties, only three farmers discussed their attitudes regarding the deficiencies in current regulations on biosecurity.

‘*But the problem is because of the lack of government control. The policy is wrong. My neighbour is next door to my farm, and he will pollute me. Why should I do better on biosecurity¿(EF, a local leader of the Poultry Association, Interview 2)*.

Two farmers complained about the government's intervention on the utility of agricultural land as Taiwan's agricultural land is insufficient.

‘*Some farmers are waiting for land speculation. The lack of funding for improving their farms is not the reason. (WB, a local leader of the Poultry Association, Interview 4)*

### Global-level (international trade)

The findings of this study revealed that the global factor plays an important role in Taiwan's on-farm biosecurity. There are three themes and three associated subthemes related to farmers' decisions about the implementation of biosecurity measures, as shown in Table 1, such as “Feed and petrol” under the theme of “Costs and profits”; “Opportunity for the export of chicken meats” under the theme of “Industry development”; “Competition for the access to the domestic market” under the theme of “Domestic market access”.

The influence of international trade cannot be ignored since the demand for imported raw materials is critical. In the study, 18 farmers mentioned their concerns about the high costs of imported feed materials and petrol. Indeed, it significantly affected domestic farmers' competitiveness with imported chicken meats. The profit opportunity via the global market is a potential motivation for more ambitious farmers in Taiwan's chicken industry. When considering the opportunity of exports, two farmers expressed positive expectations and attitudes while one had a pessimistic view.

## Discussion

The case of Taiwan is unique. Due to high-density intensive poultry production, although biosecurity guidelines have been delivered to farmers for two decades, avian influenza H5 strains have circulated in Taiwan's farms since 2012. The results of this study support our argument that multi-faceted barriers influence the adoption of on-farm biosecurity, and understanding the determinants of farmers' biosecurity mindset from the farmers' viewpoints provides a major opportunity for the government to achieve wide adoption of biosecurity measures within the chicken sector.

In this study, preliminary data was gathered first-hand, directly from Taiwan's farmers to provide a deeper understanding of determinants of farmers' biosecurity mindset. Biosecurity practises at the farm level involve complex factors such as attitudes, disease status and various considerations incorporating agriculture, society and economics. These factors influence farmers' decision-making about biosecurity. As Heffernan et al. (4) and Enticott and Wilkinson (60) argued, scientific evidence and economic concerns are not the only reasons why farmers consider the adoption of on-farm biosecurity, farmers' knowledge and perceptions of disease risks rely on their information sources, experiences and culture.

According to our findings, high costs, chicken diseases (particularly avian influenza), and the practicalities of government biosecurity regulations and policies are the key issues at the core of these operational challenges. Since chicken production makes a major contribution to household income for many local farmers (63), farmers have to consider economic aspects when the implementation of biosecurity increases production costs (2). In this study, the interviewed farmers allocated resources for the implementation of biosecurity practises based on their justification for the optimisation of resource utilisation. Although the farmers were reluctant to provide business-related information, in particular profit or income information, they reported that biosecurity measures, particularly vaccines, count for 5-8% of the total cost. Egg farmers also indicated that their willingness to undertake biosecurity-related measures will be negatively influenced by the government's control policy for egg prices.

Social structures associated with farming communities and responsibility for disease outbreaks is a mature area of knowledge in the literature (22, 64–67). Gates et al. (68) demonstrated that farmers' underreporting behaviours may be due to negative attitudes towards control measures or distrust in government authorities. Our findings align with existing literature which illustrates the importance of disease reporting by neighbouring farms for disease management and biosecurity (64, 67). Additionally, we discovered the determinant of neighbours' attitudes towards farm hygiene and their acceptance of the existing farms which accords with the findings of Alabi et al. (69) that neighbours' complaints against chicken farms as residential houses are located close to chicken houses. When rural landscapes become multifunctional, it is challenging to share biosecurity responsibility in local communities (70). Although this study focused on factors related to farmers' biosecurity mindset, our research adds value to current knowledge by supporting what the literature says.

Recent studies have examined the influence of farmers' attitudes and perceptions towards disease risks on their adoption of biosecurity. Many studies illustrated that farmers' perception of controllable disease risks is a key determinant (4, 57, 71). However, when farmers consider a disease uncontrollable, Enticott (72) and Shortall et al. (15) demonstrated that luck will be the alternative. Similar to those studies, our findings support their arguments: some of Taiwan's farmers perceive the risks of AID controllable while some take a different view of those risks: uncontrollable, and the notion of “luck/ fatalism” emerges. However, we surprisingly discovered that few farmers in Taiwan develop another alternative: the intention to ignore the perceived risks. Those farmers considered AID a common cold and even some of them intended to immunise their chickens by natural infection. The difference in farmers' risk perception and risk management may be due to the circulation of avian influenza strains in Taiwan. They believed AID vaccines can effectively prevent their chickens from circulating AID, but the government prohibited the use of AID vaccines. Further study is required to understand how their farms at high risk are more vulnerable to AID infection and to design more effective communication strategies in countering their misbehaviours.

Barclay (57), Gunn et al. (62), and Naylor et al. (73) demonstrated that farmers in the UK and Australia point out that the government should take major responsibility for biosecurity. Alternatively, they believe the government should make a greater contribution. In addition, Shortfall et al. (15) discovered that cattle farmers in the UK do not have a “blame culture”, and Naylor et al. (73) supported that poultry farmers are less likely to blame the government in England. In contrast to their findings, we discovered that Taiwan's farmers accept taking major responsibility for disease management. Nevertheless, in the context of unpredicted risks for AID, they reject the government's intentions to ask farmers to take major responsibility for the outbreaks of AID, and they blame both the government's regulations and employees. Taiwan's farmers have little confidence in the attitudes and motivations of government employees, leading to disappointment with government policy and government plans to change traditional production patterns. According to farmers' accounts, they perceived the motivations of government employees as being uninterested or reluctant to look for benefits for farmers. Furthermore, government interventions for the public's good may induce conflict between farmers and the government. For example, the government's slaughter bans for improving public health brought significant disruptions experienced throughout the chicken value chain. Although this intervention was considered not directly related to biosecurity, it induced farmers' negative views of the government and also distorted farmers' willingness to improve biosecurity. The findings are likely to be helpful in shaping future government interventions which may be related to chicken producers.

### Individual-level

In this study, farmers freely shared their experiences and opinions regarding the difficulties of conducting on-farm biosecurity to prevent diseases. Our findings align with existing literature which reports that farmers lack trust in their governments (74–76) resulting in complaints about the impracticality of the current biosecurity policy. Authors such as Rong (77) and Zawojska (79) have investigated the importance of farmers' trust in their respective governments. More specifically, authors like Palmer et al. (58) and Maclean et al. (78) have sought to understand the effect of trust on farmers' responses to the outbreak of diseases within farming systems and the adoption of biosecurity respectively. The former revealed that the government's economic rationalism makes some farmers feel unsupported; the latter indicated that the adoption of biosecurity can be improved if farmers trust their government and government-related scientific institutions.

Through further analysis of the subthemes, in contrast to the literature, Taiwan's farmers are receptive to the biosecurity information provided by the government; however, they distrust government employees. Our study has determined that the government needs to establish better relationships with farmers, based on trust, as well as with other relevant stakeholders. The participating farmers often discussed the importance of government employees' attitudes when considering the adoption of biosecurity policy. Our findings suggest that farmers have difficulty trusting the government and feel unjustly manipulated or restricted by government employees and policies.

In addition, the lack of trust in the government may also be due to inconsistent definitions of biosecurity among stakeholders. Enticott (71, 80) and Bingham et al. (81) stated that although biosecurity concepts have been widely discussed, there is no generally agreed definition. Wilkinson et al. (82) revealed that in the UK there is no legal basis for many biosecurity activities undertaken or recommended since only cleaning and disinfection are regulated by laws. Policymakers, scientists, veterinarians, farmers, and the public are very likely to have diverging perceptions in relation to what biosecurity means and what its usefulness is. If stakeholders see biosecurity problems in different ways, they may have different thinking about biosecurity-related solutions. For example, Shortall et al. (15) discovered that veterinarians and farmers have different framings in terms of biosecurity resulting in the limited success of biosecurity. Veterinarians frame biosecurity problems at individual and interpersonal levels that diseases can be controlled by individual farmers working with them; however, farmers consider biosecurity problems uncontrollable due to logistical, economic and geographical factors.

Typically, people define what biosecurity means based on their own experience and knowledge which then leads to widely differing interpretations of the biosecurity concept (60). Heider (83) suggested that people's needs and cognitive biases often distort their perceptions of causality. As a complex array of factors affects the effectiveness of biosecurity, there will be bias and errors in explaining and identifying the most critical biosecurity measures. When farmers who hardly implement biosecurity practises have never experienced an AID outbreak, it can be difficult to provide convincing evidence to persuade such farmers to implement biosecurity measures. In addition, since the implementation of biosecurity measures related to government policy cannot wholly prevent disease outbreaks, negative opinions about biosecurity have been developed among farmers after they justified the effort. Some farmers expressed fatalism or intended to ignore the perceived risks of AID when considering the effectiveness of biosecurity. Farmers' extremely negative attitude towards disease control may perhaps be further explained as “learned helplessness” based on the attribution theory in clinical psychology (84). Farmers who regard disease as an uncontrollable natural event may attribute disease outbreaks to the Will of God; therefore, biosecurity measures are likely to be perceived as ineffective for disease prevention by these types of farmers. For the underlying mechanisms of bias or errors, attribution theory can provide another explanation (85). Self-serving bias is a common type of attributional bias amongst the public (86) which can be used to explain why farmers tend to attribute disease outbreaks to external, unstable and uncontrollable factors. As a result, those farmers with negative views in relation to the effectiveness of on-farm biosecurity may have little trust in governments. An example of this was the 2013 outbreak of H7N9 avian influenza in Taiwan. When the government introduced a slaughter ban in traditional markets in an attempt to protect human health, there were significant disruptions experienced throughout the chicken value chain (87). Despite the government's best efforts, resource inefficiencies resulted in widespread complaints as government policy was seen as unworkable to people in the industry. Paradoxically, an alarming mismatch seems to exist between the government's work and farmers' need for workable solutions. Policymakers and scientists need to work together for practical solutions to gain farmers' trust and ease farmers' concerns (88). Our findings suggest that mutual trust and close collaboration need to be re-established through a full understanding of and timely response to farmers' needs and expectations with a long-term approach.

Farmers with adequate knowledge of the importance of biosecurity were more likely to take up activities aimed at preventing the introduction and transmission of infectious diseases. In addition, being aware of the negative consequences of the effects of disease outbreaks on income generation was likely to motivate farmers to adhere to biosecurity requirements. Having acquired biosecurity knowledge, coupled with experience working in the industry, formed a basis for the adoption of biosecurity measures. Important to note from the findings is that most of the biosecurity knowledge acquired was because of the training programmes provided by the local groups and the Poultry Association in Taiwan. Most of those programmes were financially supported by the government sector. Biosecurity education, skills training and resource provision need to be delivered continuously in support of sustainable biosecurity behaviours. Considerable time and efforts are required to improve and maintain biosecurity-related measures and facilities. Although the participants did not express their needs for any cost-effectiveness analysis in the subtheme of ‘The utility of research’, the information provided in a cost-effectiveness analysis of individual farms can be beneficial for farmers to evaluate the potential economic benefits of biosecurity systems.

### Group-level

Participation of farmers and farmer groups is of major interest, and the sustainability of biosecurity requires the consideration of the needs of farmers and farmer groups in addition to the effort they put into achieving these needs. Through effective participation and dialogue with farmers and farmer groups in the stage of policy development, positive responsiveness to the policy can be maintained over time. Our findings suggest a lack of trust amongst the participants and their neighbouring farmers. Targeted education programmes for the farmers and their neighbouring farmers can reduce conflicts and promote cooperative and supportive relationships.

Although the participants complained about their neighbouring farmers, they hardly mentioned peer pressure from farmer groups. Most farmers appreciated farmers' groups and felt supported through the provision of training courses in relation to biosecurity. Dione et al. (89) suggested that, when farmer groups demonstrate their commitment to on-farm biosecurity, individual farmers' group membership positively influences their acceptance of biosecurity. Using role models in a local group, who have benefited from the implementation of biosecurity practises, can provide evidence for biosecurity promotion. In addition, a social network approach can be applied to more effectively transmit positive attitudes in relation to biosecurity to other network members by identifying farmers' multidimensional network relationships such as opinion leaders and centrally-located individuals.

### Organisation-level

Taiwan's inherent challenges, for example, its subtropical climate and limited availability of farmland, have resulted in a dominance of the high density of chicken farms with intensive chicken rearing systems. Worryingly, production problems have been exacerbated in recent years because of climate change and the increasing costs of imported feed ingredients (37, 39). In western Taiwan, most farms are very close together (39, 90), resulting in potential risks for infectious pathogens spread not only within local areas but also to other parts of the country. Infectious diseases are transmitted through a variety of means, such as vehicles for chicks or feed (91). There were 1,144 outbreaks of H5 subtype highly pathogenic notifiable avian influenza (HPAI) between 2012 and 2017 (92), and at least five types of H5 strains circulated in Taiwan's farms (93). Intervention strategies at this level can be designed to provide supportive materials, for example, by increasing the supply of vaccines and medication and strengthening the surveillance systems.

Most egg farmers in Taiwan have no selling direct channel but mainly rely on middlemen to access the market. Li (37) suggests that it only takes a couple of farmers to develop their channels by marketing their products independently or through transporters; we suggest that this is an attractive consideration for chicken producers in Taiwan. The increasing intensification of the competitiveness of Taiwan's domestic chicken market (37, 39) has led farmers to become agricultural entrepreneurs, investing in brand development and advertising channels. On the other hand, some farmers remain parochial and are adamant that the government should provide support for sales and marketing. Considering these factors, better access to domestic markets may stimulate farmers' motivation to devote themselves to improving their farm management and biosecurity. Market pricing interventions from the government may provide smallholders with a secure way to respond to increased market competition. Conversely, as Tsakok (94) suggests, since costs mainly affect the effectiveness of competition, it might likewise cause smallholders to become less competitive. In developing countries, when farming transitions from a government-based marketing system to a private, premium marketing system, farmers must adapt to the changing situation. As a profit-oriented focus makes farmers more entrepreneurial, a dual effort must be made to take advantage of economies of scale to produce high quantities of chicken while also focusing on the production of products with high food safety and food quality. In any case, farmers likewise face various difficulties. For example, Patrick (95) demonstrated that farmers experience issues with transport to markets or new technologies. Even when they have opportunities to enter a new market, price fluctuations prohibit a steady cash flow. In this way, smallholders intensify their farming yet in addition experience higher risks of losing animals due to disease invasion. As such, biosecurity-related knowledge, skills and technologies can help farmers' decision-making process in their pursuit to increase profits (96). For example, vaccines can lessen the chance of disease transmission, and the application of modern evaporative cooling systems can improve production performance (39).

### Community-level

The social-ecological model demonstrated the complex interplay amongst the range of factors. Due to avian influenza outbreaks, the public attributed the cause of these outbreaks to farmers. Although the farmers accept taking the major responsibility for the implementation of on-farm biosecurity, they rejected the government's ask farmers to take major responsibility for the cause of these outbreaks. This may result in the farmers' lack of trust in the government. Government employees' active engagement in risk communication with the public may improve the relationships amongst chicken farmers, government employees and the public.

In addition, intervention strategies at this level can be designed to change the social norms such as by helping build local brands to increase the consumers' confidence in local brands. Broad societal factors related to a public culture in which on-farm biosecurity is encouraged can be included. For example, the growing demand by the public for products with high food safety and food quality may be an opportunity for the chicken industry to reform the value chain. That is, if farmers expand their local market access, their income will increase, and as a result, their willingness to invest in biosecurity will increase.

### Government-level

With the factors investigated at each level, the social-ecological model developed in this study can further lead to reviews of the current policies adopted by communities, organisations, or industries (97). The government should try to implement more transparent communication and policies to increase farmers' trust in the government, as the efforts could be helpful in the promotion of on-farm biosecurity. This research has highlighted the importance of linking epidemiology and social science research for the improvement of on-farm biosecurity and the control of animal diseases. Evidence-based knowledge about the complex web of factors influencing on-farm biosecurity in this study can inform policymakers and scientists to reconsider strategies for working with farmers which will then improve on-farm biosecurity.

Taking into consideration the large numbers of smallholders in Taiwan who operate relatively simple chicken production units and are thus easily adaptable to a changing environment, the government needs to provide more flexible biosecurity-related provisions to the industry. Industry-oriented interventions are also needed such as utilising risk-based assessment to identify critical control points for individual farms according to their existing management tools.

### Global-level (international trade)

The findings of the current study revealed that international trade has an important, unique influence on farmers' decisions associated with on-farm biosecurity. Taiwan's farmers are concerned that costs and profits will be affected by imported feed, petrol and chicken meats. They also expect to export chicken meats in accordance with international standards in food safety and disease management. The chicken industry policy aims to reduce the impact of global competition and enhance the competitive ability of local producers. The price of imported chicken meats is lower than domestic chicken meats, resulting in higher consumption of imported meat. Despite the fact that there has been no urgent need to exploit the global market, the expansion of market access may incentivise farmers to improve the effectiveness of disease control and the standards of food safety on individual farms. Intervention strategies at this level can include improving global market access opportunities for local producers and promoting local brand identity in the domestic market.

This research did not provide details on how to integrate behaviour change theories into biosecurity policy development, but the following points are important in achieving behaviour change resulting in more effective on-farm biosecurity:

- Collaboration amongst all stakeholders: It is not unusual for stakeholders to have different interests and conflicts and interpret relevant situations from their own viewpoints. It is impossible to achieve long-term biosecurity behaviour changes at the farm level without government support (13). It is time to be a step back within the relationship between those farmers and the government, and the understanding of government officials' viewpoints is another important topic for further study for the success of biosecurity systems.
- Science-based knowledge generated by behavioural change theories: Although exploratory factors related to farmers' biosecurity mindset have been identified in this research, it is unclear how those factors will affect groups of farmers or individual farmers and how those factors will interact with each other or affect other components of the production process. More studies need to be conducted in the field to confirm the underlying causes of biosecurity behaviour changes and evaluate the effectiveness of behaviour change interventions (88, 98).
- One Health approach and interdisciplinary research: The One Health approach provides a platform for the integration of social science and epidemiology to conduct interdisciplinary research. This approach will be critical in further study to understand the root causes of infectious diseases at the farm level and address the gap in farmers' attitudes and behaviours in relation to on-farm biosecurity. More interdisciplinary studies need to be conducted to find workable solutions.

Finding consensus views was not our objective. Instead, we aimed to explore the breadth and depth of chicken farmers' attitudes to adopting biosecurity practises. Semi-structured interviews directly explore farmers' views in-depth about on-farm biosecurity. The interviews placed the farmers being studied at the heart of the study process. Thus, we studied the farmers and understood their farming lives. Face-to-face interviews were particularly powerful in gaining in-depth insight into the context of farmers' farming lives and their individual attitudes towards biosecurity; this was far more effective and efficient than focus groups or surveys. Qualitative content analysis was chosen based on its major advantage of researchers being able to determine disparate meanings of data based on their field experience. This flexibility is helpful in creating meaning from a range of similar views that are communicated by participants through different choices of words.

In this study, the theoretical saturation of the data was reached (*n* = 25). The study population included commercial chicken farmers from a variety of flock sizes and farm types. Backyard farmers were excluded due to there being very few of these farmers and the focus of the study primarily being on commercial chicken production. In Taiwan, most farms are run as family businesses; thus, farmworkers were not included in the study. Convenience sampling was chosen due to the limited accessibility of participants. The small sample size of the study means that caution needs to be exercised when it comes to the representativeness of the conclusions for all chicken farmers in Taiwan. The farmers interviewed were all the farm owners. Although some of them hired workers, they all engaged with the day-to-day running of the farm and husbandry practises. Pao (99) reported that broiler farmers' education and age have a positive association with their biosecurity practises. Because the farmers in the sample are more educated than the average farmer in Taiwan, the sample may be biassed towards farmers with more positive biosecurity attitudes than the average farmer who is less educated and therefore likely to have a less-developed sense of biosecurity needs. Further research is required to explore Taiwan's chicken farmers' attitudes towards biosecurity through confirmatory research.

In conclusion, this study discovered that many factors such as the context and infrastructure of the external environment, individual farmers' characteristics and instinct factors, costs incurred for individual farmers to change their current biosecurity behaviours and animal disease status are relevant to farmers' biosecurity behaviour options. Those complex factors determined the adoption of biosecurity (88). To the best of our knowledge, the application of the social-ecological model with six levels to construct determinants of farmers' biosecurity mindset, in which global effects are taken into consideration, is still uncommon. The findings support our argument that multilevel efforts are needed to understand for the promotion of on-farm biosecurity systems. The study may aid in the design of biosecurity interventions to manage risks in relation to infectious diseases, which are important to the wider society. Starting from this study, future studies could examine the multilevel impacts on farmers' biosecurity decision-making process in a larger population sample. The novelty of this research lies in its wider relevance to Taiwan's chicken production industry in that it provides first-hand evidence-based knowledge to demonstrate a wide number of determinants that construct farmers' biosecurity mindset. With regard to some factors that overlap and interconnect with the others across multiple levels, the findings of this study also highlight the importance of systems thinking for the development of behavioural interventions. This social-ecological model based on systems thinking allows the development of animal health management approaches that are tailored to the local farm level (100). Farmers' viewpoints revealed the impacts of multifaceted, complex barriers on on-farm biosecurity. The relationship between the occurrence of chicken diseases and on-farm biosecurity was an important focus of discussion among the farmers. Furthermore, the practicalities of government policy seemed to be of critical concern. For the sustainability of biosecurity, promoting farmers' engagement lies in building trust amongst individual farmers, neighbouring farmers and the government. Due to the relative scarcity of literature, these findings may be of wider relevance to chicken producers in Taiwan and the wider geographic region and identify areas that can be targeted by policymakers to improve production efficiency and effectiveness of on-farm biosecurity measures, resulting in chicken products of improved food safety. The six-level social-ecological model developed in this study explains the complicated influence of macro socio-economic conditions on farmers' biosecurity mindset and provides potential solutions for the challenges associated with improving on-farm biosecurity through systems thinking. This model allows adaptation to the local context and should result in more effective animal health management at the farm level.

## Data availability statement

The original contributions presented in the study are included in the article/Supplementary material, further inquiries can be directed to the corresponding author.

## Ethics statement

The studies involving human participants were reviewed and approved by Ethics and Welfare Committee of the Royal Veterinary College, University of London, the United Kingdom (approval #URN 2014 0116H). Written informed consent for participation was not required for this study in accordance with the national legislation and the institutional requirements.

## Author contributions

H-nP did the research and writing of the article as part of her Ph.d. thesis. DP, EJ, and WS were H-nP's supervisors and were involved extensively throughout all processes of the research and writing of this article. T-sY provided support in collecting data and developing the critiqued aspects of data analysis. J-sT provided the critiqued aspects of data analysis and presentation. All authors have approved the article submitted for publication.

## Conflict of interest

The authors declare that the research was conducted in the absence of any commercial or financial relationships that could be perceived as a potential conflict of interest.

## Publisher's note

All claims expressed in this article are solely those of the authors and do not necessarily represent those of their affiliated organisations, or those of the publisher, the editors and the reviewers. Any product that may be evaluated in this article, or claim that may be made by its manufacturer, is not guaranteed or endorsed by the publisher.

## Abbreviations

AID: avian influenza disease
HPAI: highly pathogenic notifiable avian influenza
LDCC: local livestock disease control centre.
