# Supplementary material for: Determinants of farmers' biosecurity mindset: A social-ecological model using systems thinking
Source: Front Vet Sci. 2022 Aug 15;9:959934. doi: 10.3389/fvets.2022.959934 (PMC9420990; doi:10.3389/fvets.2022.959934)
Supplement: Supplementary file 1 [file Data_Sheet_1.pdf]

Determinants of farmers' biosecurity mindset: A social-ecological model using systems thinking

Hai-ni Pao[*1], Elizabeth Jackson[*2], Tsang-sung Yang[3], Jyan-syung Tsai[4], Watson H.T. Sung[5] , Dirk U. Pfeiffer[1,6]

[1]Veterinary Epidemiology, Economics and Public Health Group, Department of Pathobiology and Population Sciences, Royal Veterinary College, Hatfield, United Kingdom

[2] School of Management & Marketing, Curtin University, Perth, Western Australia, Australia

[3]Independent Researcher

[4] Department of Finance and Cooperative Management, National Taipei University, Taiwan.

[5]Agricultural Bank of Taiwan

[6]Centre for Applied One Health Research and Policy Advice, Jockey Club College of Veterinary Medicine and Life Sciences, City University, Hong Kong SAR, China

*Correspondence:

Haini Pao, paohaini@gmail.com

Present address: No 5, Lane 57, Rose Road, Shing-tien District, New Taipei City, Taiwan (R.O.C.)

Supplementary Information

Table S.1 The comparison of the themes and sub-themes revealed (in the study) with the literature

Level	Theme	Sub-theme	Reference
Individual (farmers and chickens)	**Chickens†**		
	**Health and welfare**		Sayers et al., 2013; Denis-Robichaud et al., 2019; Aleri & Laurence, 2020
	**Diseases†**		
		Endemic, epidemic or exotic diseases†	Valeeva et al., 2011; Heffernan et al., 2008; Moya et al.,2020; Suit-B et al., 2021
		Disease process	Heffernan et al., 2008; Brennan et al., 2016
	**Farmers and farms†**		
	**Attitudes†**		
		Willingness/ Little interest in biosecurity, disease prevention and control†	Gunn et al., 2008; Barclay, 2005; Enticott, 2008a; Heffernan et al., 2008; Ellis-Iversen et al., 2010
		Farmers' attitudes and perceptions towards disease risks † (e.g., perceived susceptibility and severity)	Enticott, 2008a and 2008b; Moore et al 2008; Valeeva et al., 2011; Garforth, Bailey, & Tranter, 2013; Brennan et al., 2016; Mankad, 2016; Mankad, 2016; Damiaans et al., 2018;Garcia, Huff, & Huff, 2020; Lestari et al., 2019; Renault et al., 2020; Suit-B et al., 2021
		Perception of controllable disease risks†	Barclay, 2005; Heffernan et al., 2008; Enticott, 2008a
		Luck /fatalism†	Enticott, 2008a;Enticott, 2016; Shortall et al.,2016
		Intention to ignore the perceived risks† (e.g. considering avian influenza a common cold and immunising chickens by natural infection)	
		Attitudes, awareness, perceptions and beliefs about biosecurity†	Delabbio et al.,2003; Delabbio et al.,2004; Delabbio et al., 2005; Casal et al., 2007; Moore et al 2008; Mankad, 2016; Delpont et al., 2020; Suit-B et al., 2021
		An underlying failure to appreciate the complex and multiple flows	Enticott, 2008c
		Underlying reasons to appreciate biosecurity†	
		Perceived benefits† (e.g., return on	Enticott, 2008c; Gunn et al., 2008; Moore et al 2008; Valeeva et

Level	Theme	Sub-theme	Reference
		investment or maintain business continuity during a disease outbreak)	al., 2011; Sayers et al., 2013; Brennan et al., 2016; Damiaans et al., 2018; Denis-Robichaud et al., 2019; Lestari et al., 2019; Wolff, Abigaba, & Lewerin, 2019; Zulbainarni & Rizky, 2019; Renault et al., 2020; Gupta et al., 2020; Suit-B et al., 2021
		Effectiveness†	Sayers et al., 2013; Brennan et al., 2016; Mankad, 2016; Damiaans et al., 2018; Renault et al., 2018; Renault et al., 2020; Royden et al., 2021
		Most desirable/useful biosecurity measures †	Heffernan et al., 2008; Renault et al., 2018; Suit-B et al., 2021
		Feasibility †	Damiaans et al., 2018; Renault et al., 2018; Pudenz, Schulz, &Tonsor, 2019<br>Suit-B et al., 2021
		Misunderstanding †	Denis-Robichaud et al., 2019
	**Ambition**†		
		Belief in self-efficacy †	Ellis-Iversen et al., 2010, Delpont et al., 2020
		Ability †	Gunn et al., 2008; Brennan et al., 2016
	**Resources**†		
		Time†	Barclay, 2005; Moore et al 2008; Brennan et al., 2016; Shortall et al., 2016; Damiaans et al., 2018; Royden et al., 2021
		Income, capital, or economic concerns†	Barclay, 2005; Eze & Okudu, 2008; Heffernan et al.2008; Enticott & Wilkinson, 2013; Laanen et al., 2014; Shortall et al., 2016; Omowon et al., 2019; Wolff, Abigaba, & Lewerin, 2019; Garcia, Huff, & Huff, 2020; Tung et al., 2020; Suit-B et al., 2021
		Costs of biosecurity practices †	Moore et al 2008; Fraser et al., 2010; Kristensen & Jakobsen; 2011; Mankad, 2016; Damiaans et al., 2018; Royden et al., 2021
		Labour†	Tung et al., 2020
		Land†	Tung et al., 2020
		Access to veterinary service†	Wolff, Abigaba, & Lewerin, 2019
		Testing accuracy†	Brennan et al., 2016
	**Trust in the government**†		

Level	Theme	Sub-theme	Reference
		Negative views to information and educational documents provided by the government	Heffernan et al., 2008; Palmer, Fozdar, & Sully, 2009; Brennan et al., 2016
		Negative views to government employees' attitudes†	Elbers et al., 2010
		A social dilemma of the trade-off between public benefits and farmers’ private interests†	Kristensen & Jakobsen; 2011; Limon et al., 2014
	**Habits, knowledge and experience†**		Cui & Liu, 2016; Young et al., 2015; Lestari et al., 2019; Wolff, Abigaba, & Lewerin, 2019; Renault et al., 2020
		The available information and sources †	Heffernan et al.2008; Enticott & Wilkinson, 2013; Gupta et al., 2020
		Private veterinarians as the preferred motivators†	Ellis-Iversen et al., 2010; Brennan et al., 2016
		The lack of information or education	Barclay, 2005; Nantima et al., 2016; Shortall et al., 2016; Dione et al., 2017
		Scientific evidence for the efficacy of biosecurity practices†	Barclay, 2005; Gunn et al., 2008; Heffernan et al.2008; Valeeva et al., 2011; Enticott & Wilkinson, 2013; Garforth, Bailey, & Tranter, 2013
		Current knowledge and experience†	Casal et al., 2007
		Previous experiences of a disease †	Enticott, 2008; Heffernan et al., 2008; Enticott & Wilkinson, 2013; Garforth, Bailey, & Tranter, 2013; Suit-B et al., 2021
		Farmers’ knowledge of diseases †	Gillespie, 2000; Sanderson, Dargatz, & Garry, 2000; O'Bryen & Lee, 2003; Enticott, 2008a and 2008b; Elbers et al., 2010; Ellis-Iversen et al., 2010
		Previous experiences of a specific measure †	Garforth, Bailey, & Tranter, 2013; Renault et al., 2018
		Reasonable measures have been done †	Garforth, Bailey, & Tranter, 2013
		Fitting into the current practices†	Garcia, Huff, & Huff, 2020; Gupta et al., 2020
	**Social status†**		
		Farming experiences†	Racicot et al., 2012; Delpont et al., 2020
		Education†	Racicot et al., 2012; Ajewole et al 2014; Dione et al., 2020; Mankad, 2016
		Age†	Ellis-Iversen et al., 2010; Sayers et al., 2013; Ajewole et al 2014; Mankad, 2016
		Sex	Mankad, 2016
	**Characteristics of farmers**		

Level	Theme	Sub-theme	Reference
		Social responsibility for food safety	Ellis-Iversen et al., 2010
		Farmers' personality	Racicot et al., 2012; Renault et al., 2020
		Guilt, shame and prejudice for disease outbreaks	Elbers et al., 2010
		Learning styles	Moore et al 2008
	**Farm type†**		
		Characteristics of the enterprise †	Garforth, Bailey, & Tranter, 2013; Royden et al., 2021
		Perceived risk to their enterprise †	Palmer, Fozdar, & Sully, 2009
		Farm size †	Ellis-Iversen et al., 2010; Sayers et al., 2013; Mankad, 2016
		Location†	Moore et al 2008; Sayers et al., 2013; Mankad, 2016; Dione et al., 2020
	**Workers' aspects**		
		Peer pressure for the accountability and job security/ Manager's commitments to biosecurity/ Education levels/ Personality/ Experiences with disease outbreaks/ Personal beliefs of biosecurity	Delabbio, 2006
Group (family, friends, neighbours and farmers' associations)	**Social culture/ pressure †**		Mankad, 2016; Lestari et al., 2019; Wolff, Abigaba, & Lewerin, 2019; Delpont et al., 2020
		Peer pressure	Wolff, Abigaba, & Lewerin, 2019
		Neighbours' attitudes† (e.g. neighbours' attitudes towards farm hygiene and their acceptance of the existing farms)	Alabi et al.,2014
	**Industry development†**		
		Cooperation and competition†	
		Neighbour farmers do not report their outbreaks†	Barclay, 2005; Garforth, Baile & Tranter, 2013; Maye & Chan, 2020
		Group membership and group culture†	Heffernan et al.2008; Enticott & Wilkinson, 2013; Dione et al., 2020
		Negative attitudes about forming groups	Heffernan et al., 2008
		Trust in farmers' community†	Heffernan et al., 2008; Palmer, Fozdar, & Sully, 2009
		Peers' knowledge, perceptions and experiences of diseases†	Enticott, 2008 and 2008a; Heffernan et al., 2008
Organisation (the chicken industry)	**Production conditions†**		
		Costs and profits†	
		Economic pressure from society and	Ellis-Iversen et al., 2010

Level	Theme	Sub-theme	Reference
		industry †	
		Weather†	Barclay, 2005
		Agricultural space and environment†	Lestari et al., 2019
		Chaotic and difficult to control † *(e.g., high density of farms*)	Enticott, 2008c
	**Domestic market access†**		
		Consumers' attitudes towards locally-produced chickens†	
		Consumers' confidence†	Ellis-Iversen et al., 2010
		Consumer demand†	Ellis-Iversen et al., 2010
		Access to the domestic market†	
		Market access channels†	Oladele, Antwi & Kolawole, 2013
	**The supply of vaccines and medication†**		
		The access to vaccines†	Brennan & Christley, 2012; Shortall et al.,2017
		The trust in vaccines and medication†	MacPhillamy et al., 2021
	**Industry development†**		
		Organisation culture (e.g. biosecurity culture)	Heffernan et al.2008; Enticott & Wilkinson, 2013; Shortall et al., 2016
		The strong power held by relevant stakeholders†	Sambo et al., 2015
		Opportunity for the export of chicken meats†	Oladele, Antwi & Kolawole, 2013
		Generation gaps and a sunset industry†	Tipples, R., 2007
Community (the public)	**Brand establishment to promote local produce†**		
		The lack of trusted domestically produced brands†	Ellis-Iversen et al., 2010
	**Public attitudes towards the poultry industry†**		
		The public's negative attitudes †	Ellis-Iversen et al., 2010; Brennan et al., 2016
		Unrealistic expectations from the public and the government† (e.g. consumers' expectations for low prices or the government's intentions to ask farmers to take major responsibility for avian influenza outbreaks)	Ellis-Iversen et al., 2010
	**Human health†**		Aleri & Laurence, 2020
Government (public policies and government	**Government intervention (biosecurity related) †**		
		Compulsory and compensation of biosecurity measures†	Sayers et al., 2013; Mankad, 2016; Omowon et al., 2019; Aleri & Laurence, 2020
		The utility of research †	

Level	Theme	Sub-theme	Reference
employees' attitudes)		*Biosecurity suggested by scientists lacks common sense and practical experiences*†	Enticott, 2008b and 2008c
		A simple cost-effectiveness analysis	Barclay, 2005
		The lack of trust and credibility in government-related scientific institutions	Enticott, 2008b; Palmer, Fozdar, & Sully, 2009; Elbers et al., 2010; Limon et al., 2014
		Not the key issue to study†	
		The lack of regulations or support†	Naylor et al., 2018
		Biosecurity should be compulsory†	Heffernan et al., 2008
		Government interventions with financial inducements or penalties	Ellis-Iversen et al., 2010; Kristensen & Jakobsen, 2011
		The practicality of government regulations†	Naylor et al., 2018
		Opposing attitudes to government intervention †	Heffernan et al.,2008
		Negative opinion on control measures †	Elbers et al., 2010
		Biosecurity suggested by the government lacks common sense and practical experiences †	Enticott, 2008b and 2008c
		Disadvantages of reporting and dissatisfaction with post-reporting procedures†	Barclay, 2005; Elbers et al., 2010
		Uncertainty and the lack of transparency in reporting procedures †	Elbers et al., 2010
		The credibility of biosecurity information provided by the government	Garforth, Bailey, & Tranter, 2013
		Negative views to government employees' attitudes†	Elbers et al., 2010
	**Responsibility**†		Renault et al., 2020
		Major responsibility belongs to the government or the government should make a greater contribution	Barclay, 2005; Gunn et al., 2008; Naylor et al., 2018
		Major responsibility belongs to farmers†	
	**Government intervention (not directly related to biosecurity)** †		
		The practicality of government regulations†	
		Market mechanisms†	

Level	Theme	Sub-theme	Reference
		The utility of agricultural land†	Sinclair, Curtis & Freeman, 2020
Global (international trade)	**Costs and profits†**		
		Feed and petrol†	Sambo et al., 2015
	**Industry development†**		
		Opportunity for the export of chicken meats†	Oladele, Antwi & Kolawole, 2013
	**Domestic market access†**		
		Competition for the access to the domestic market†	Oladele, Antwi & Kolawole, 2013

† Themes/ sub-themes found in this study.

Table S.2 Overview of farmers interviewed

Characteristics	Farmers (n, (%))
**Gender**	25(100%)
Female	0 (0%)
Male	25 (100%)
**Farm management experiences**	25(100%)
Less than 10 years	0 (0%)
10-19 years	7 (28%)
More than 20 years	18 (72%)
**A group leader of the Poultry Association**	25(100%)
Yes	13(52%)
No	12(48%)
**Farm type**	25(100%)
Egg farm (EF)	10(40%)
White-chicken broiler farm (WB)	8 (32%)
Indigenous chicken farm (IC)	7 (28%)
**Farm size**	25(100%)
>50 thousand	9(36%)
Between 20 and 50 thousand	10(40%)
Between 3 thousand and 20 thousand	4(16%)
<3 thousand	2(8%)
**Farm location**	25(100%)
Northern	10(40%)
Central	8(32%)
Southern	6(24%)
Eastern	1(4%)

Appendix 1: Interview guide:

A. Explanation

1. **Appreciate** the participant for taking part
2. Explain that content form and **ensure** there will be no possibility to link the participant's name or address to the analysis results (**No coding** either).
3. Promise the **final report** of the project will be provided to them via the Poultry Association
4. If the participant does not want to be **recorded**, it shall be omitted.
5. Introduce **the purpose** of this study and my role.
6. Ask the participant to **sign the content form.**

B. General overview

Farm type - Let's start with a general overview of your farm

1. How many animals do you have in your farm (including chicken and other livestock and pets)?

Prompts:

- ☐ Production capacity? Method and frequency?
- ☐ Getting smaller or larger? Why?

Farmer – Can you tell me some things about yourself?

2. How long have you or your family been involved in this farm?

Prompts:

- ☐ Why do you choose this type of chicken?
- ☐ Change chicken breeds in the past 2 years? Why?

3. What makes it difficult to be a farmer? Have you faced **any big challenge/ disaster** happened during the past 2 years?

Prompts:

- ☐ What happened?
- ☐ **Disease?** Poultry disease/ food borne/zoonotic

Biosecurity – Let me ask you a few questions about disease…

4. Which poultry **diseases,** for example bird flu, Salmonella, are particular threats to chicken farmers **in your region**? If there is any other diseases that have affected the flock(s)?

Prompts:

- ☐ Why?
- ☐ How do they do to reduce the threat?
- ☐ How do you do to reduce the threat?

5. Tell me about what you do to manage disease

Prompts:

- [ ] **What measure(s)**? Good enough?
- [ ] How may **budget, manpower and time** used for biosecurity?
- [ ] Change measures in these two years? Why?
- [ ] Do you have any **disease** outbreak? What happened?

Resource

6. Is there anything that **restricts you from doing a better job** of managing disease outbreaks on your farm?

Prompts:

- [ ] What resource do you need?
- [ ] Any external resource you can obtain? How to get?

Surrounding

7. Is there any farm close to your farm(e.g. chicken, duck, pig.)? Can you tell me about the **neighbouring farms** and if you think they cause any disease concern?

Prompts:

- [ ] Chicken, duck, pig
- [ ] Farm type and the distance
- [ ] Why concerned? Any **disease outbreak** in the past 2 years? How serious?

8. What about migratory birds or wild animals? Do you think they could be a disease threat to your farm?

Prompts:

- [ ] What kinds of animals/species?
- [ ] How often and where do they approach your farm (e.g.: daily, monthly or seasonally)?
- [ ] How do you do to reduce their contact?

Other

9. Is there anything else that you think would be important about on-farm biosecurity?

Well, that's the end of the interview. Thank you very much for your time and participating in this research. The next step is for me to continue talking to chicken farmers about their on-farm disease management and then I've got the large task ahead of me of analysing all of this interview data.

My name is Haini PAO and you can contact me at anytime about the interview. My E-mail is hpao@rvc.ac.uk; my cell phone No: +886-983286582;+44-7473306936

References:

Ajewole, O.C., & Akinwumi, A.A. (2014) Awareness and practice of biosecurity measures in small scale poultry production in Ekiti State, Nigeria. *IOSR Journal of Agriculture and Veterinary Science*. 7 (11) Ver. I, 24-29.

Alabi, R., Aghimien, C., Osasogie, D., & Erie, O. (2014) Environmental effects of poultry production in Edo State, Nigeria. *Journal of Experimental Agriculture International*, 4(12), 1773-1782. https://doi.org/10.9734/AJEA/2014/11958

Aleri, J.W., & Laurence, M. (2020) A description of biosecurity practices among selected dairy farmers across Australia. *Animal Production Science* [online], available: https://doi.org/10.1071/AN19340

Barclay, E. (2005) *Local community preparedness for an emergency animal disease outbreak- A report for the rural industries research and development*. Rural Industries Research and Development Corporation, Australian government.

Brennan, M.L., & Christley, R.M. (2012) Biosecurity on cattle farms: A study in north-west England. *PLoS One*, 7(1), e28139. https://doi.org/10.1371/journal.pone.0028139

Brennan, M.L., Wright N., Wapenaar W., Jarratt S., Hobson-West P., Richens I.F., Kaler J., Buchanan H., Huxley J.N., & O'Connor, H.M. (2016) Exploring attitudes and beliefs towards implementing cattle disease prevention and control measures: A Qualitative study with dairy farmers in Great Britain. *Animals* (Basel), 6(10), 61. https://doi.org/10.3390/ani6100061

Casal, J., De Manuel, A., Mateu, E., & Martín, M. (2007) Biosecurity measures on swine farms in Spain: perceptions by farmers and their relationship to current on-farm measures. *Preventive Veterinary Medicine*, 82(1), 138-150. https://doi.org/10.1016/j.prevetmed.2007.05.012

Cui, B., & Liu, Z.P. (2016) Determinants of knowledge and biosecurity preventive behaviors for highly pathogenic avian influenza risk among Chinese Poultry farmers. *Avian Diseases*, 60(2), 480–486. https://doi.org/10.1637/11361-010116-reg

Damiaans, B., Sarrazin, S., Heremans, E., & Dewulf, J. (2018) Perception, motivators and obstacles of biosecurity in cattle production. *Vlaams Diergeneeskundig Tijdschrift*, 87, 150-163. https://doi.org/10.21825/vdt.v87i3.16079

Delabbio, J. (2006) How farm workers learn to use and practice biosecurity. *Journal of Extension*, 44(6) [online], available: http://www.joe.org/joe/2006december/a1.php

Delabbio, J.L., Johnson, G.R., Murphy, B.R., Hallerman, E., Woart, A., & Mcmullin, S.L. (2005) Fish disease and biosecurity: Attitudes, beliefs, and perceptions of managers and owners of commercial finfish recirculating facilities in the United States and Canada. *Journal of Aquatic Animal Health*, 17(2), 153-159. https://doi.org/10.1577/H04-005.1

Delabbio, J.L., Murphy, B.R., Johnson, G.R., & Hallerman, E.M. (2003) Characteristics of the recirculation sector of finfish aquaculture in the United States and Canada. *International Journal of Recirculating Aquaculture*, 4, 5-23. http://hdl.handle.net/10919/90590

Delabbio, J.L., Murphy, B.R., Johnson, G.R., & McMullin, S.L. (2004) An assessment of biosecurity utilization in the recirculation sector of finfish aquaculture in the United States and Canada. *Aquaculture,* 242(1-4), 165-179.

Delpont, M., Racicot, M., Durivage, A., Fornili, L., Guerin, J.-L., Vaillancourt, J.-P., & Paul, M.C. (2020) Determinants of biosecurity practices in French duck farms after a H5N8 highly pathogenic avian influenza epidemic: The effect of farmer knowledge, attitudes and personality traits. *Transboundary and Emerging Diseases*, 68(1), 51-61. https://doi.org/10.1111/tbed.13462

- Denis-Robichaud, J., Kelton, D.F., Bauman, C.A., Barkema, H.W., Keefe, G.P., & Dubuc, J. (2019) Canadian dairy farmers' perception of the efficacy of biosecurity practices. *Journal of Dairy Science*, 102 (11), 10657-10669. https://doi.org/0.3168/jds.2019-16312

Dione, M.M., Dohoo, I., Ndiwa, N., Poole, J., Ouma, E., Amia, W.C., & Wieland, B. (2020) Impact of participatory training of smallholder pig farmers on knowledge, attitudes, and practices regarding biosecurity for the control of African swine fever in Uganda. *Transboundary and Emerging Diseases*, 67(6), 2482-2493. https://doi.org/10.1111/tbed.13587

Dione, M.M., Akol, J., Roesel, K., Kungu, J., Ouma, E.A., Wieland, B., & Pezo, D. (2017) Risk factors for African swine fever in smallholder pig production systems in Uganda. *Transboundary and Emerging Diseases*, 64(3), 872– 882. https://doi.org/10.1111/tbed.12452

Elbers, A.R.W., Gorgievski-Duijvesteijn, M.J., Zarafshani, K., & Koch, G. (2010) To report or not to report: A psychosocial investigation aimed at improving early detection of avian influenza outbreaks. *Scientific and Technical Review of the Office International des Epizooties* (Paris), 29(3), 435-449. https://doi.org/10.20506/rst.29.3.1988

Ellis-Iversen, J., Cook, A.J., Watson, E., Nielen, M., Larkin, L., Wooldridge, M., & Hogeveen, H. (2010) Perceptions, circumstances and motivators that influence implementation of zoonotic control programs on cattle farms. *Preventive Veterinary Medicine*, 93(4), 276-285. https://doi.org/10.1016/j.prevetmed.2009.11.005

Enticott, G., & Wilkinson, K. (2013) Biosecurity: Whose knowledge counts? In: Dobson, A., Barker K., Taylor. S. L., eds. *Biosecurity: The Socio-Politics of Invasive Species and Infectious Diseases*. Abingdon: Routledge.

Enticott, G. (2008a) Biosecurity, 'sound science' and the prevention paradox: Farmers' understandings of animal health [Working Paper], *BRASS Working Paper Series,* 44. Cardiff: BRASS, Cardiff University.

Enticott, G. (2008b) The ecological paradox: Social and natural consequences of the geographies of animal health promotion. *Transbehaviours of the Institute of British Geographers*, 33(4), 433-446. http://dx.doi.org/10.1111/j.1475-5661.2008.00321.x

Enticott, G. (2008c) The spaces of biosecurity: Prescribing and negotiating solutions to bovine tuberculosis. Environment and Planning A, 40(7), 1568-1582. http://dx.doi.org/10.1068/a40304

Enticott, G. (2016) Market instruments, biosecurity and place-based understandings of animal 869 disease. *Journal of Rural Studies***,** 45, 312–319. https://doi.org/10.1016/j.jrurstud.2016.04.008

Eze, C.I., & Okudu, P.O. (2008) Discriminant analysis of poultry farmers technology adoption potentials in Abia State Nigeria. *Global Approaches to Extension Practice: A Journal of Agricultural Extensio*n, 4, 2. http://dx.doi.org/10.4314/gaep.v4i2.43318

Fraser, R.W., Williams, N.T., Powell, L.F., & Cook, A.J. (2010) Reducing Campylobacter and Salmonella infection: Two studies of the economic cost and attitude to adoption of on-farm biosecurity measures. *Zoonoses Public Health*, 57(7-8), e109-115. https://doi.org/10.1111/j.1863-2378.2009.01295.x

Garcia, J.D., Huff, A.G., & Huff, E.S. (2020) Understanding farmers' biosecurity beliefs and behaviours related to antimicrobial resistant bacteria in Michigan. *USA. Animal Production Science,* 60(5), 674-682. https://doi.org/10.3168/jds.2020-19614

Garforth, C.J., Bailey, A.P., & Tranter, R.B. (2013) Farmers' attitudes to disease risk management in England: A comparative analysis of sheep and pig farmers. *Preventive Veterinary Medicine*, 110(3-4), 456-466. https://doi.org/10.1016/j.prevetmed.2013.02.018

Gillespie, J.R. (2000) The underlying interrelated issues of biosecurity. *Journal American Veterinary Medicine Association*, 216(5), 662-664. https://doi.org/10.2460/javma.2000.216.662

Gunn, G., Heffernan, C., Hall, M., McLeod, A., & Hovi, M. (2008) Measuring and comparing constraints to improved biosecurity amongst GB farmers, veterinarians and the auxiliary industries. *Preventive Veterinary Medicine*, 84(3-4), 310-323. https://doi.org/10.1016/j.prevetmed.2007.12.003

Gupta, S. D., Fournié, G., Hoque, M. A., & Henning, J. (2020) Factors influencing chicken farmers' decisions to implement prevention and control measures to reduce avian influenza virus spread under endemic conditions. *Transboundary and Emerging Diseases*, 68(1), 194-207. https://doi.org/10.1111/tbed.13757

Heffernan, C., Nielsen, L., Thomson, K., & Gunn, G. (2008) An exploration of the drivers to bio-security collective action among a sample of UK cattle and sheep farmers. *Preventive Veterinary Medicine*, 87 (3), 358-372. https://doi.org/10.1016/j.prevetmed.2008.05.007

Kristensen, E., & Jakobsen, E.B. (2011) Danish dairy farmers' perception of biosecurity. *Preventive Veterinary Medicine*, 99(2), 122-129. https://doi.org/10.1016/j.prevetmed.2011.01.010

Laanen, M., Maes, D., Hendriksen, C., Gelaude, P., De Vliegher, S., Rosseel, Y., & Dewulf, J. (2014) Pig, cattle and poultry farmers with a known interest in research have comparable perspectives on disease prevention and on-farm biosecurity. *Preventive Veterinary Medicine*, 115, 1-9. https://doi.org/10.1016/j.prevetmed.2014.03.015

Lestari, V.S., Rahardja, D.P., Mappigau, P., Rohani, S.T., & Sirajuddin, S.N. (2019) Beef cattle farmers behavior toward biosecurity. *Journal of the Indonesian Tropical Animal Agricultur*e, 44(2), 204-212. http://dx.doi.org/10.14710/jitaa.44.2.204-212

Limon, G., Lewis, E.G., Chang, Y., Ruiz, H., Balanza, M.E., & Guitian, J. (2014) Using mixed methods to investigate factors influencing reporting of livestock diseases: A case study among smallholders in Bolivia. *Preventive Veterinary Medicine*, 113(2), 185-196. http://dx.doi.org/10.1016/j.prevetmed.2013.11.004

MacPhillamy, I., Olmo, L., Young, J., Nampanya, S., Suon, S., Khounsy, S., Windsor, P., Toribio, J.A., & Bush, R. (2021) Changes in farmer animal health and biosecurity knowledge, attitudes and practices: Insights from Cambodia and Laos. *Transboundary and Emerging Diseases*, early view, https://doi.org/10.1111/tbed.14328

Mankad A. (2016) Psychological influences on biosecurity control and farmer decision-making. A review. *Psychology Agronomy for Sustainable Development*, 36, 40. https://doi.org/10.1007/s13593-016-0375-9

Maye, D., & Chan, K.W.R. (2020) On-farm biosecurity in livestock production: farmer behaviour, cultural identities and practices of care. *Emerging Topics in Life Sciences*, 4(5), 521-530. https://doi.org/10.1042/ETLS20200063

Moore, D.A., Merryman, M.L., Hartman, M.L., & Klingborg, D.L. (2008) Comparison of published recommendations regarding biosecurity practices for various production animal species and classes. *Journal of the American Veterinary Medical Association*, 233 (2), 249-256. https://doi.org/10.2460/javma.233.2.249

Moya S., Tirado F., Espluga J., Ciaravino G., Armengol R., Diéguez J., Yus E., Benavides B., Casal J., & Allepuz A. (2020) Dairy farmers' decision-making to implement biosecurity measures: A study of psychosocial factors. *Transboundary and Emerging Diseases*, 67(2), 698-710. https://doi.org/10.1111/tbed.13387

Nantima, N., Davies, J., Dione, M., Ocaido, M., Okoth, E., Mugisha, A., & Bishop, R. (2016) Enhancing knowledge and awareness of biosecurity practices for control of African swine fever among smallholder pig farmers in four districts along the Kenya-Uganda border. *Tropical Animal Health and Production*, 48, 727– 734. https://doi.org/10.1007/s11250-016-1015-8

Naylor, R., Hamilton-Webb, A., Little, R., & Maye, D. (2018) The 'good farmer': Farmer identities and the control of exotic livestock disease in England. *Sociologia Ruralis*, 58 (1), 3-19. https://doi.org/10.1111/soru.12127

O'Bryen, P.J., & Lee, C.S. (2003) Discussion summary on biosecurity in aquaculture production systems: Exclusion of pathogens and other desirables. In: Lee, C.S., O'Bryen, J. P., eds. *Biosecurity in Aquaculture Production Systems: Exclusion of Pathogens and Other Desirables*. Baton Rouge, Louisiana: The World Aquaculture Society.

Oladele, O.I., Antwi, M.A., & Kolawole, A. (2013) Factors Influencing Demand for Animal Health Services and Knowledge of Biosecurity Among Livestock Farmers Along Border Villages of South Africa and Namibia. *The Journal of Applied Research in Veterinary Medicine*, 11(2), 123-129. https://www.jarvm.com/articles/Vol11Iss2/Vol11%20Iss2VETOladele.pdf

Omowon, A.A., Daodu, O.B., Omowon, A.M., & Bello, I.I. (2019) Knowledge, attitude and practices of pig farmers post African swine fever outbreaks in Ogun and Oyo states of Nigeria. *Sokoto Journal of Veterinary Sciences*, 17(4), 14-24. https://doi.org/10.4314/sokjvs.v17i4.3

Palmer, S., Fozdar, F., & Sully, M. (2009) The effect of trust on west Australian farmers' responses to infectious livestock diseases. *Sociologia Ruralis*, 49(4), 360-374. https://doi.org/10.1111/j.1467- 9523.2009.00495.x

Pao, H.N. (2017) Identifying human behavioural and epidemiological factors critical for the success of a high biosecurity compartmentalisation scheme in Taiwan's broiler industry (PhD Thesis). Royal Veterinary College, University of London.

Pudenz, C.C., Schulz, L.L., &Tonsor, G.T. (2019) Adoption of secure pork supply plan biosecurity by U.S. swine producers. *Frontiers in Veterinary Science*, 6. https://www.frontiersin.org/article/10.3389/fvets.2019.00146

Racicot, M., Venne, D., Durivage, A., & Vaillancourt, J.P. (2012) Evaluation of the relationship between personality traits, experience, education and biosecurity compliance on poultry farms in Quebec, Canada. *Preventive Veterinary Medicine*, 103(2), 201-207. https://doi.org/10.1016/j.prevetmed.2011.08.011

Renault V., Damiaans B., Humblet M.F., Jiménez Ruiz S., García Bocanegra I., Brennan M.L., Casal J., Petit E., Pieper L., Simoneit C., Tourette I., van Wuyckhuise L., Sarrazin S., Dewulf J., & Saegerman C. (2020) Cattle farmers' perception of biosecurity measures and the main predictors of behaviour change: The first European-wide pilot study. *Transboundary and Emerging Diseases*. 68(6), 3305-3319. https://doi.org/10.1111/tbed.13935.

Renault, V. Damiaans, B. Sarrazin, S., Humblet M.-F., Dewulf, J., & Saegerman, C. (2018) Biosecurity practices in Belgian cattle farming: Level of implementation, constraints, and weaknesses. *Transboundary and Emerging Diseases*, 65(2), 1-16. https://doi.org/10.1111/tbed.12865

Royden, A., Christley, R., Prendiville, A., & Williams, N.J. (2021) The role of biosecurity in the control of Campylobacter: A qualitative study of the attitudes and perceptions of UK broiler farm workers. *Frontiers in Veterinary Science, 8*. https://www.frontiersin.org/article/10.3389/fvets.2021.751699

Sambo, E., Bettridge, J., Dessie, T., Amare, A., Habte, T., Wigley, P., & Christley, R.M. (2015) Participatory evaluation of chicken health and production constraints in Ethiopia. *Preventive Veterinary Medicine*, 118(1), 117-27. https://doi.org/10.1016/j.prevetmed.2014.10.014

Sanderson, M.W., Dargatz, D.A., & Garry, F.B. (2000) Biosecurity practices of beef-cow calf producers. *Journal of American Veterinary Medical Association*, 217(2), 185-189. https://doi.org/10.2460/javma.2000.217.185

Sayers, R.G., Sayers, G.P., Mee, J.F., Good, M., Bermingham, M.L., Grant, J., & Dillon, P.G. (2013) A survey investigating implementation of, and opinions towards, biosecurity measures on 2 commercial Irish dairy farms. *The Veterinary Journal*, 197(2), 259-267. http://dx.doi.org/10.1016/j.tvjl.2012.11.017

Shortall, O., Green, M., Brennan, M., Wapenaar, W., & Kaler, J. (2017) Exploring expert opinion on the practicality and effectiveness of biosecurity measures on dairy farms in the United Kingdom using choice modeling. Journal of Dairy Science, 100(3), 2225-2239. https://doi.org/10.3168/jds.2016-11435

Shortall, O., Ruston, A., Green, M., Brennan, M., Wapenaar, W., & Kaler, J. (2016) Broken biosecurity? Veterinarians' framing of biosecurity on dairy farms in England. *Preventive Veterinary Medicine*, 132, 20-31. https://doi.org/10.1016/j.prevetmed.2016.06.001

Sinclair, K., Curtis, A., & Freeman, P. (2020) Biosecurity in multifunctional landscapes: challenges for approaches based on the concept of 'shared responsibility'. *Preventive Veterinary Medicine*, 178, 104682. https://doi.org/10.1016/j.prevetmed.2019.04.011

Suit-B, Y., Hassan, L., Krauss, S.E., Ooi, P.T., Ramanoon, S.Z., Yasmin, A.R., & Epstein, J.H. (2021) Mental model of Malaysian Pig farmers in implementing disease prevention and control practices. *Frontiers in Veterinary Science*, 8. https://www.frontiersin.org/article/10.3389/fvets.2021.695702

Tipples, R. (2007) The Further Re-regulation of Farming Employment Relations in New Zealand. *Sociologia Ruralis*, 47 (1), 63-79. https://doi.org/10.1111/j.1467-9523.2007.00428.x

Tung, D.X., Tuan, H.A., Minh, N.T.T., & Padungtod, P. (2020) Economic analysis of enhanced biosecurity practices in three types of chicken farms in Northern Vietnam. *Livestock Research for Rural Development*, 32 (4) [online], available: http://www.lrrd.org/lrrd32/4/xuanto32054.html (accessed 17 June 2020)

Valeeva, N.I., van Asseldonk, M.A., & Backus, G.B. (2011) Perceived risk and strategy efficacy as motivators of risk management strategy adoption to prevent animal diseases in pig farming. *Preventive Veterinary Medicine*, 102(4), 284-295. https://doi.org/10.1016/j.prevetmed.2011.08.005

Wolff, C., Abigaba, S., & Lewerin, S.S. (2019) Ugandan cattle farmers' perceived needs of disease prevention and strategies to improve biosecurity. B*MC Veterinary Research*, 15(1), 208. https://doi.org/10.1186/s12917-019-1961-2

Young, J.R., Evans-Kocinski, S., Bush, R.D., & Windsor, P.A. (2015) Improving smallholder farmer biosecurity in the mekong region through change management. *Transboundary and Emerging Diseases*, 62(5), 491– 504. https://doi.org/10.1111/tbed.12181

Zulbainarni, N., & Rizky, L. (2019) Cost of biosecurity application: Comparing aquaculture system and fish health in traditional fish farm. International Society for Economics and Social Sciences of Animal Health - South East Asia 2019 (ISESSAH-SEA 2019). *Advances in Health Sciences Research*, 19, 98-99. https://dx.doi.org/10.2991/isessah-19.2019.27
